# The activity of the C4-dicarboxylic acid chemoreceptor of *Pseudomonas aeruginosa* is controlled by chemoattractants and antagonists

**DOI:** 10.1038/s41598-018-20283-7

**Published:** 2018-02-01

**Authors:** David Martín-Mora, Álvaro Ortega, Francisco J. Pérez-Maldonado, Tino Krell, Miguel A. Matilla

**Affiliations:** Department of Environmental Protection, Estación Experimental del Zaidín, Consejo Superior de Investigaciones Científicas, Granada, Spain

## Abstract

Chemotaxis toward organic acids has been associated with colonization fitness and virulence and the opportunistic pathogen *Pseudomonas aeruginosa* exhibits taxis toward several tricarboxylic acid intermediates. In this study, we used high-throughput ligand screening and isothermal titration calorimetry to demonstrate that the ligand binding domain (LBD) of the chemoreceptor PA2652 directly recognizes five C4-dicarboxylic acids with *K*_D_ values ranging from 23 µM to 1.24 mM. *In vivo* experimentation showed that three of the identified ligands act as chemoattractants whereas two of them behave as antagonists by inhibiting the downstream chemotaxis signalling cascade. *In vitro* and *in vivo* competition assays showed that antagonists compete with chemoattractants for binding to PA2652-LBD, thereby decreasing the affinity for chemoattractants and the subsequent chemotactic response. Two chemosensory pathways encoded in the genome of *P. aeruginosa*, *che* and *che2*, have been associated to chemotaxis but we found that only the *che* pathway is involved in PA2652-mediated taxis. The receptor PA2652 is predicted to contain a sCACHE LBD and analytical ultracentrifugation analyses showed that PA2652-LBD is dimeric in the presence and the absence of ligands. Our results indicate the feasibility of using antagonists to interfere specifically with chemotaxis, which may be an alternative strategy to fight bacterial pathogens.

## Introduction

A series of different signal transduction systems permit bacteria to sense changing environmental conditions and to generate adaptive responses. Next to one- and two-component systems, chemosensory pathways represent a major mechanism in bacterial signal transduction^1–3^. In these systems, the direct binding of chemoeffectors or chemoeffector-loaded periplasmic binding proteins to the ligand binding domain (LBD) of chemoreceptors^4^ generates a molecular stimulus that alters the autophosphorylation of the histidine kinase CheA and consequently the transphosphorylation of the CheY response regulator, which represents the pathway output^2^. Chemosensory pathways were shown to mediate chemotaxis and type IV pili-based motility, or are involved in regulating alternative cellular processes^5–7^.

The opportunistic pathogen *Pseudomonas aeruginosa* is an important model organism to investigate chemosensory pathways^8^. Its chemoreceptors feed into 4 different pathways. Two of these signalling cascades, *che* and *che2*, mediate flagellum-mediated taxis^9,10^. However, the *wsp* pathway controls c-di-GMP levels^5^ whereas the fourth pathway, *chp*, is responsible for type IV pili-mediated motility^11,12^ and the regulation of cAMP levels^13^. The function of most of the 26 *P. aeruginosa* chemoreceptors remains unknown but others have been characterized in depth, including the three paralogous receptors PctA, PctB and PctC for the chemotaxis to different amino acids^14–17^ and the CtpH and CtpL^18,19^ receptors that mediate chemoattraction to inorganic phosphate.

*P. aeruginosa* is a ubiquitous pathogen able to infect a broad range of different hosts such as human, animals, plants or fungi^20^. Part of our research interests consists in assessing how chemosensory signalling mechanisms compare in phylogenetically related species that have different lifestyles. To address this issue we study *P. putida* KT2440, a non-pathogenic and nutritionally versatile soil bacterium with saprophytic lifestyle^21–23^. The genome of *P. putida* KT2440 encodes 3 chemosensory pathways^24^ and 27 chemoreceptors, which is very similar to the number of chemoreceptors in *P. aeruginosa* PAO1. However, sequence analyses and functional data appear to indicate that these are not sets of homologous proteins with homologous function. Initial evidence suggests that chemoreceptors that mediate responses to different compound classes are rather different. One such example are chemoreceptors of KT2440 and PAO1 for tricarboxylic acid (TCA) cycle intermediates. In KT2440, three receptors, McpS, McpQ and McpR, have been shown to mediate responses to TCA cycle intermediates. McpS is a broad ligand range chemoreceptor that binds most of the TCA cycle intermediates^25,26^. Interestingly, McpS binds citrate, an abundant compound in plant tissues and root exudates, with only low affinity. However, McpS does not bind the metal ion complexed form of citrate^27^, which is the primary form of citrate in the environment. This may have been the reason for the evolution of McpQ, a chemoreceptor that binds specifically citrate in both its metal-free and metal-complexed forms^28^. In addition, McpR was found to mediate chemotaxis to malate and fumarate^29^. Remarkably, McpS and McpQ possess an helical bimodular (HBM) type sensor domain^30^ whereas McpR has a 4-helix bundle (4HB) domain^31^.

TCA cycle responsive chemoreceptors so far identified in *P. aeruginosa* are McpK, mediating specific responses to α-ketoglutarate^32^, as well as the malate specific receptor, PA2652^33^. Whereas McpK has an HBM type LBD, PA2652 possess a sCACHE domain. CACHE domains are abundant sensor domains in chemoreceptors and sensor kinases^34,35^ and exist in two forms: (i) sCACHE (single CACHE), composed of a single structural module; and (ii) dCACHE (double CACHE), consisting of two CACHE modules in tandem. *P. aeruginosa* and *P. putida* contain a significant number of dCACHE containing chemoreceptors, namely 5 and 9, respectively. However, the genomes of both strains only encode a single sCACHE domain containing receptor. McpP, the sCACHE containing receptor of KT2440, was found to bind acetate, pyruvate, propionate and L-lactate^36^. McpP and PA2652 share 37% of sequence identity whereas the identity of their respective LBDs is only 23% (Supplementary Fig. S1), underling the important sequence divergence.

Here we report the characterization of the chemoreceptor PA2652 of *P. aeruginosa*. The study that reported its initial identification showed that a mutant in this gene did not respond to malate^33^, but it is unknown whether it binds malate directly or via periplasmic binding proteins. This study also demonstrated that PA2652 is involved in the response of *P. aeruginosa* to malate but not to other organic acids such as succinate, 2-oxoglutarate, citrate or acetate^33^. We used here high throughput approaches^37,38^ to define more precisely the chemoeffector range of PA2652.

## Results

### PA2652 binds several C2-substituted C4-dicarboxylic acids

To identify the LBD of PA2652, its sequence was analysed by the DAS transmembrane region prediction algorithm^39^. The receptor was found to possess two transmembrane regions (Supplementary Fig. S2) and the DNA fragment encoding the section in between both regions was cloned into an expression vector. The resulting protein, PA2652-LBD, was expressed in *Escherichia coli* and purified from the soluble fraction of the *E. coli* lysate by metal affinity chromatography.

**Figure 1:**
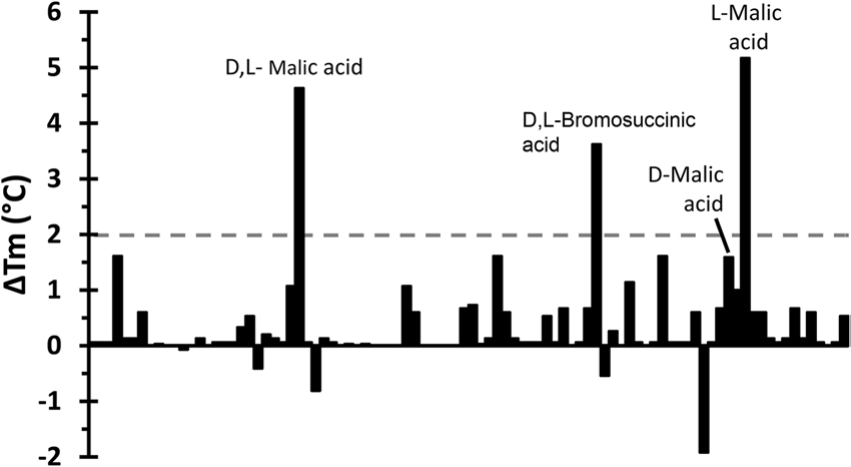
Differential Scanning Fluorimetry based high-throughput ligand screening of PA2652-LBD. Shown are Tm changes for each of the 95 compounds present in the Biolog PM1 compound array of carbon sources with respect to the Tm of the ligand-free protein of 45.5 °C. The dashed line indicates the threshold of 2 °C for significant hits.

To identify ligands that may bind to the LBD of PA2652, we conducted Differential Scanning Fluorimetry (DSF) based high throughput ligand screening assays as described previously^37,38^. DSF analyses permit the determination of melting temperature (Tm) values, which corresponds to the temperature at which half the protein is in its native conformation whereas the remaining half has undergone thermal unfolding. Since ligand binding typically enhances the thermal stability of proteins, increases in the Tm in the presence of ligands may be indicative of specific binding. We screened 450 different compounds available in five different ligand arrays from Biolog (Supplementary Fig. S3). The screened collection included different carbon and nitrogen sources, phosphorous and sulfurous compounds as well as different nutrient supplements. DSF assays evidenced a Tm of 45.5 °C for the ligand-free PA2652-LBD and Fig. 1 shows the Tm changes produced by the presence of each of the 95 compounds of the PM1 array of different carbon sources. Tm increases of more than 2 °C, an accepted threshold for significant hits, were observed for a mixture of L- and D-malic acid, D,L-bromosuccinic acid as well as L-malic acid, whereas D-malic acid did not cause significant increases. Screening of other arrays also resulted in several hits, including citraconic acid and racemic mixtures of citramalic acid (Supplementary Table S1).

**Figure 2:**
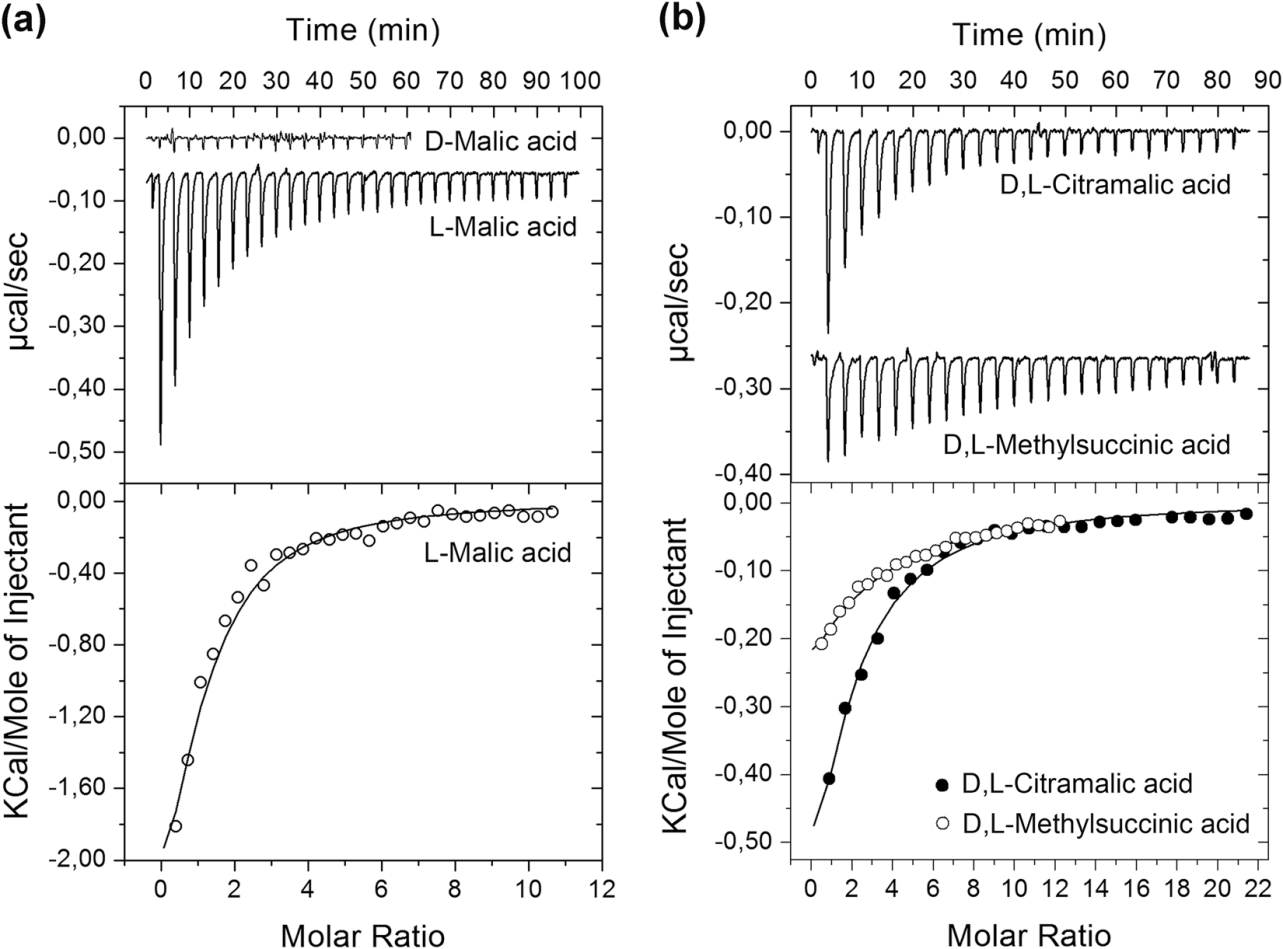
Isothermal titration calorimetry analysis of ligand binding to PA2652-LBD. (**a**) Titration with both malic acid isomers. (**b**) Titration with racemic mixtures of citramalic and methylsuccinic acids. The upper panels are the titration raw data for the injection of 9.6–14.4 μl aliquots of 1–2 mM ligand solution into 20–35 μM of protein. The lower panels are the integrated, dilution heat corrected and concentration normalized peak areas fitted with the “One binding site” model of ORIGIN.

Thermal shift assays indicate but do not constitute proof of binding^32,38^. To unambiguously determine ligand binding, we conducted Isothermal Titration Calorimetry (ITC)^40^ binding studies with the purified protein. Figure 2a shows the microcalorimetric titration of PA2652-LBD with the L- and D-isomers of malic acid. L-malic acid bound with a *K*_D_ value of 23 ± 1 µM, which is very similar to the affinities of ligands for McpP-LBD^36^. Binding was driven by both favourable enthalpy (Δ*H* = −4.2 ± 1.2 kcal) and entropy changes (*T*Δ*S* = 2.1 ± 1 kcal/mol). In marked contrast, D-malic acid did not show binding (Fig. 2a) confirming the DSF data (Fig. 1).

**Figure 3:**
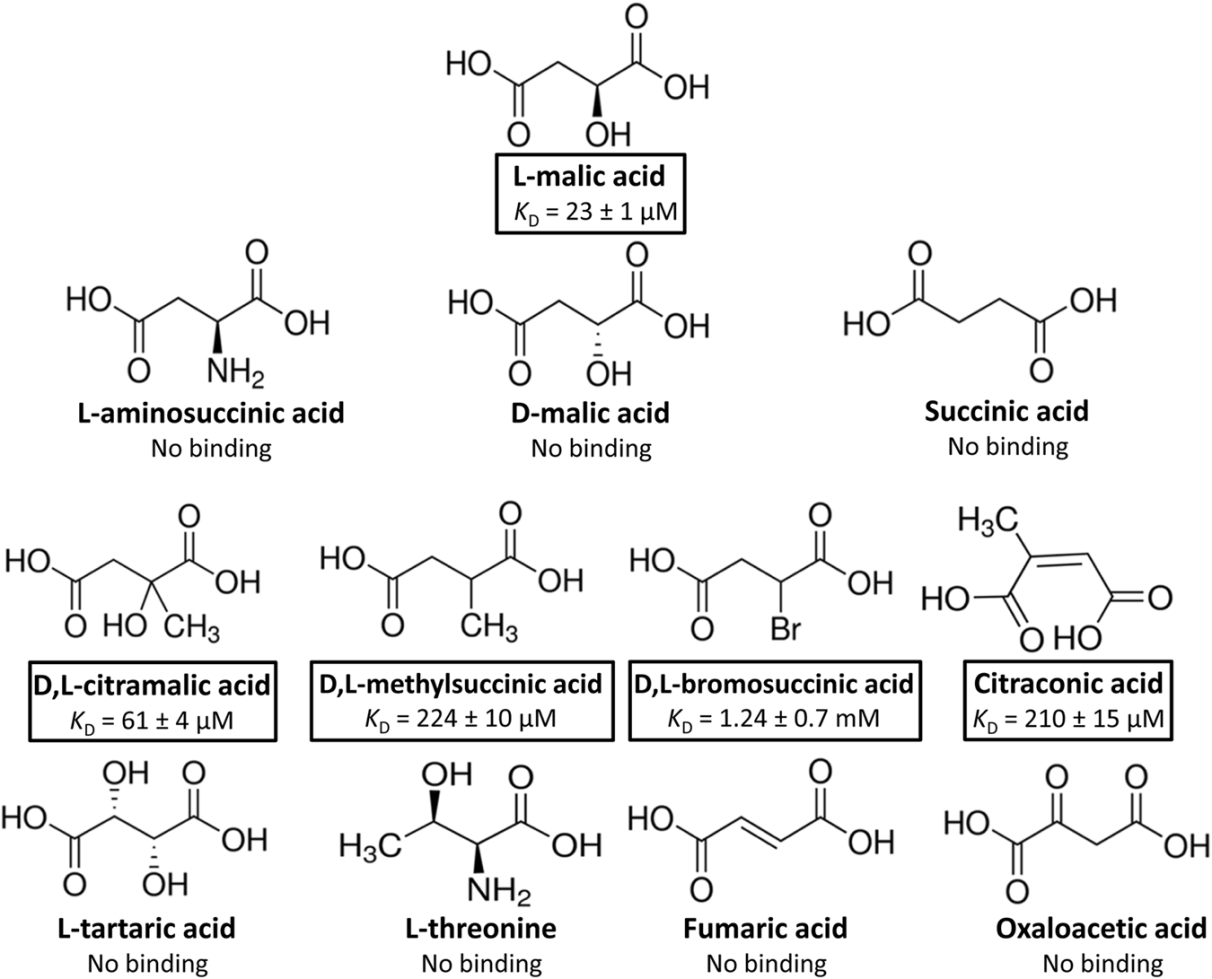
Summary of isothermal titration calorimetry studies. Shown are the structures of the five ligands that showed binding as well as of other compounds that were analysed but did not reveal binding. Dissociation constants are means and standard deviations from three experiments.

We subsequently verified by ITC the binding of other compounds that caused Tm increases of at least 2 °C and detected binding for citraconic acid and racemic mixtures of citramalic and bromosuccinic acids with *K*_D_ values of 210 ± 15 µM, 61 ± 4 µM and 1.24 ± 0.7 mM, respectively (Figs 2b and 3). As a result, it became clear that receptor PA2652 binds different C4-dicarboxylic acids and, in order to complete the ligand profile of this receptor, we analysed the binding of additional structurally related compounds. These experiments resulted in the detection of binding for D,L-methylsuccinic acid (not present in the compound arrays; Fig. 2b) whereas assays using other C4-dicarboxylates (Supplementary Table S1) such as succinic, fumaric, oxalacetic, aminosuccinic or tartaric acids resulted in an absence of binding. We also investigate whether different commercially available D- stereoisomers of citramalic and methylsuccinic acids bind to PA2652-LBD. As observed for D-malic acid, no binding was observed (Supplementary Fig. S4), as an indication that the LBD of PA2652 specifically binds L-stereoisomers of organic acids. Importantly, the five ligands recognized by PA2652 are thus C2-substituted C4-dicarboxylic acids (Fig. 3).

### L-malic acid binding does not change the dimeric state of PA2652-LBD

Ligand-induced dimerization of the chemoreceptor ligand binding domain was proposed to be a necessary prerequisite for signalling^41^. Chemoreceptors employ different types of LBD^4^ and previous studies have assessed the effect of ligand binding to the oligomeric state of individual LBDs. These experiments have resulted in two different scenarios. On one hand, individual 4HB or HBM domains are primarily monomeric in its ligand-free state and ligand binding induces dimerization^25,32,42,43^. In contrast, dCACHE domains were found to be monomeric in the absence and presence of ligands^15^. However, no information is available on the effect of ligand binding on the oligomeric state of sCACHE domains.

**Figure 4:**
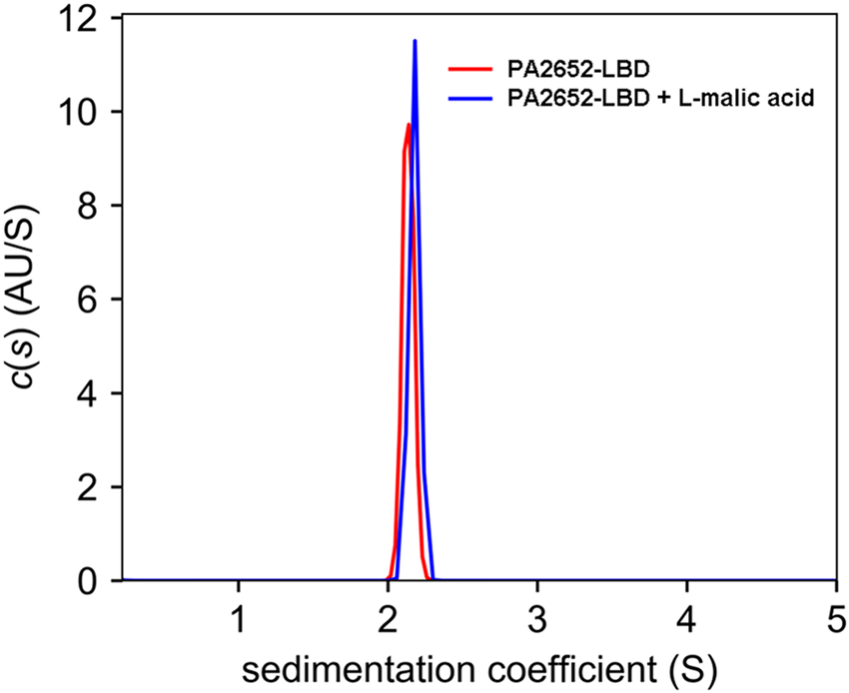
Sedimentation velocity analytical ultracentrifugation analysis of PA2652-LBD. The sedimentation coefficient profile is shown for the protein at 20 µM in the absence and in presence of 1 mM L-malic acid. Values shown are expressed at the conditions of the experiment, namely at a temperature of 7 °C and PIPES buffer.

To address this issue, PA2652-LBD was analysed by sedimentation velocity analytical ultracentrifugation (AUC). Initial experiments were conducted using concentrations of ligand-free protein ranging from 5 to 20 µM. These assays showed no significant differences in the sedimentation coefficients calculated for the species identified, which rules out hydrodynamic non-ideality behaviour. The analysis of single species in the sedimentation profile resulted in a standard sedimentation coefficient of *s*_*w,20*_ = 3.1 S and a frictional ratio of 1.4; the latter indicative of an elongated protein shape (Fig. 4). The molecular weight extracted from the sedimentation coefficient and the shape was of 41 kDa. Considering that the sequence-derived mass of the PA2652-LBD monomer is 21.5 kDa, the observed species is clearly a protein dimer. Since no shift to higher sedimentation coefficients was measured with increasing protein concentrations (data not shown), the dimer species can be considered stable over the protein concentration range analysed. The above experiments were also performed in the presence of saturating concentrations of L-malic acid. In these assays, the behaviour of PA2652-LBD was highly similar to that of the unliganded protein and data analysis resulted in the same sedimentation coefficient and frictional ratio as observed for the ligand-free protein (Fig. 4). It can therefore be concluded that PA2652-LBD forms stable dimers in the ligand-free state and that the binding of L-malic acid does not have any significant effects on the oligomerization state of the protein.

### PA2652 ligands act as attractants and antagonists

To evaluate the physiological relevance of the ligands identified, we first addressed the question of whether they could support bacterial growth as sole carbon source. To this end, we conducted growth experiments of the wild type (wt) strain and a mutant defective in *PA2652* in minimal medium supplemented with the different ligands as sole carbon sources. As shown in Supplementary Fig. S5, L-malic acid and racemic mixtures of citramalic and methylsuccinic acids were able to efficiently sustain growth of both PAO1 strains. Alternatively, D,L-bromosuccinic acid poorly supported bacterial growth, whereas citraconic acid could not be used as sole carbon source (Supplementary Fig. S5).

**Figure 5:**
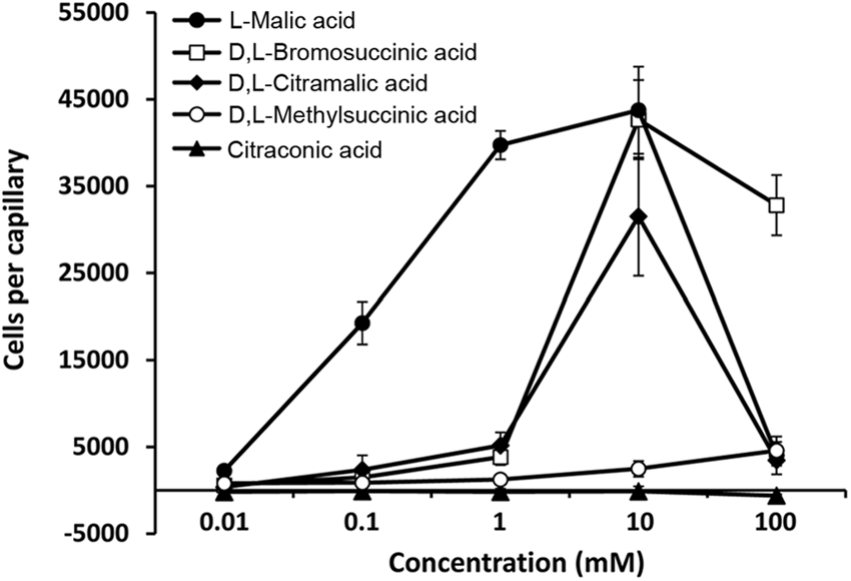
Quantitative capillary chemotaxis assays of *Pseudomonas aeruginosa* PAO1 toward different organic acids. Data are means and standard deviations from three biological replicates conducted in triplicate. Data were corrected with the number of cells that swam into buffer containing capillaries (1713 ± 231).

Secondly, we assessed the capacity of the ligands identified to induce chemotaxis. To this end, we carried out quantitative capillary chemotaxis assays using the wt strain and PA2652 ligands at concentrations ranging from 10 µM to 100 mM. Our results showed that L-malic acid induces strong chemotaxis responses over the concentration range of 100 µM to 10 mM (Fig. 5). Significant responses were also observed for racemic mixtures of bromosuccinic and citramalic acids, with a maximum chemotactic response at a concentration of 10 mM. Minor but statistically significant responses were also observed for D,L-methylsuccinic acid, whereas citraconic acid, the only ligand that did not support growth (Supplementary Fig. S5), did not cause any chemotactic response (Fig. 5).

**Figure 6:**
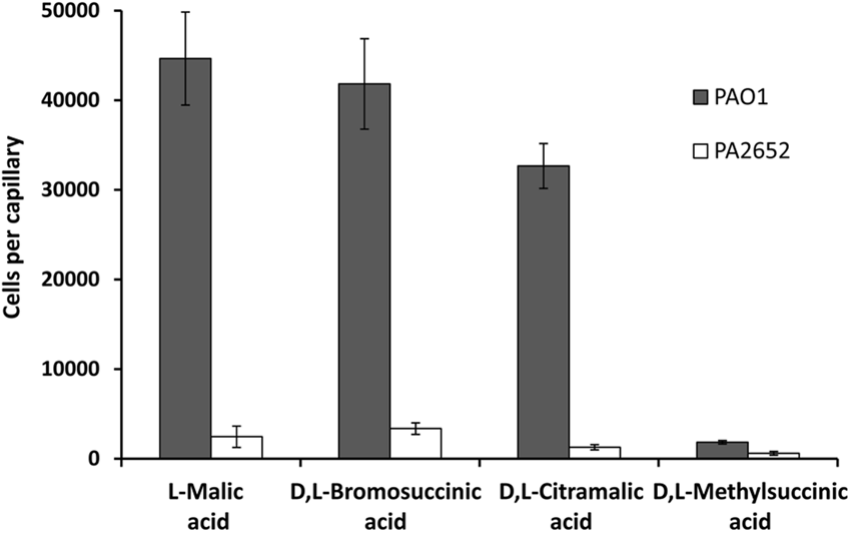
Quantitative capillary chemotaxis assays of *Pseudomonas aeruginosa* PAO1 and its mutant defective in *PA2652* to different PA2652 chemoeffectors. In all cases, chemoeffectors were used at a final concentration of 10 mM. Data were corrected with the number of cells that swam into buffer containing capillaries (1565 ± 327). Data are means and standard deviations from three biological replicates conducted in triplicate.

To determine the role of the PA2652 receptor in the observed tactic responses, we characterized phenotypically a mutant defective in the corresponding gene. Initial control experiments involved the measurement of chemotaxis of the wt and a *PA2652* mutant strain toward casamino acids, which is mediated by the three paralogous receptors PctA, PctB and PctC^14,15^. Both the wt and the mutant strain showed similar responses to casamino acids (Supplementary Fig. S6) indicating that the mutation of *PA2652* did not result in any undesired secondary effects. Quantitative chemotaxis assays of the mutant strain toward 10 mM ligand solutions showed a dramatic reduction in chemotaxis for all ligands (Fig. 6), indicating that PA2652 is the primary receptor for these C4-dicarboxylic acids. The minor responses in the mutant strain may be potentially due to a secondary receptor. Importantly, *in trans* expression of PA2652 in a mutant defective in *PA2652* resulted in the complementation of the chemotaxis defect toward C4-dicarboxylic acids (Supplementary Fig. S7).

### Attractants and antagonists compete for binding at PA2652-LBD *in vitro* and *in vivo*

**Figure 7:**
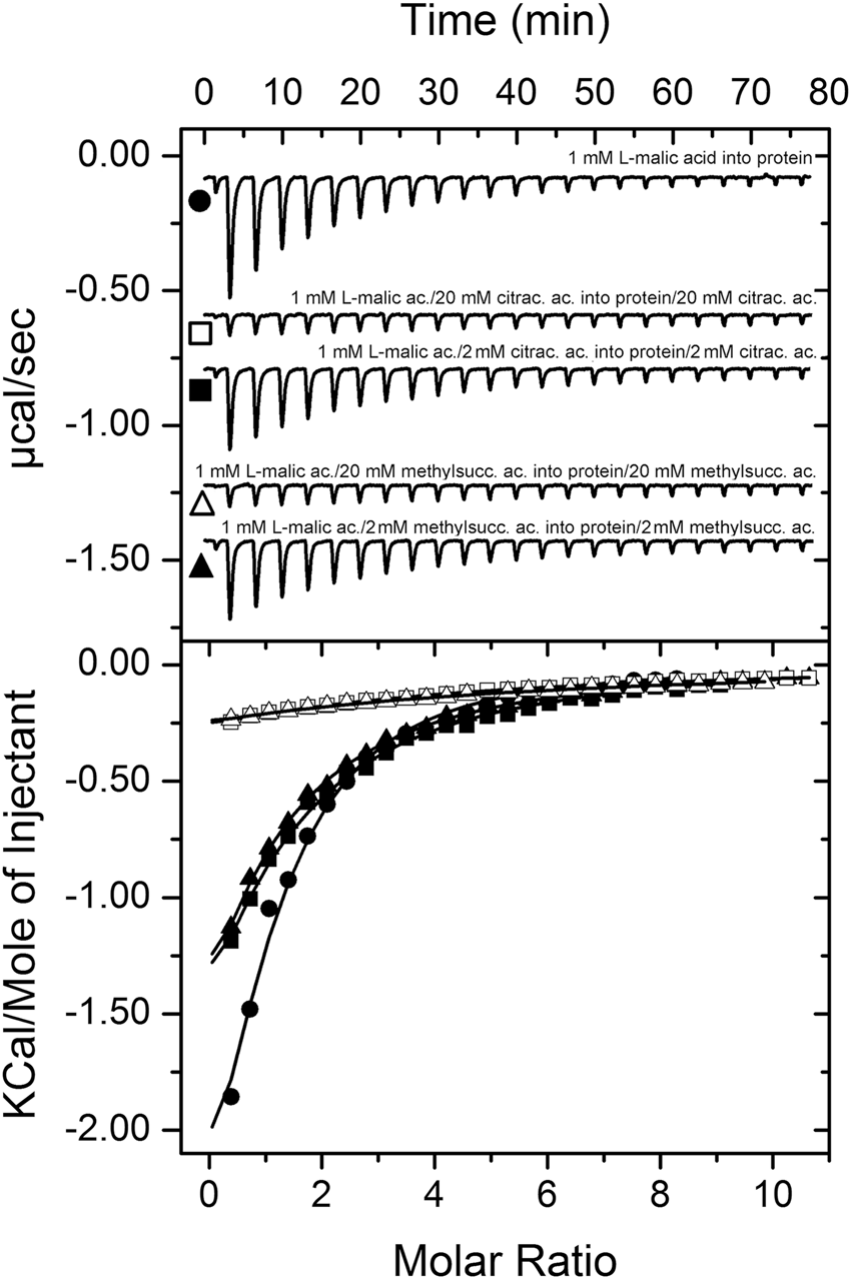
Attractants and antagonists compete for binding at PA2652-LBD *in vitro*. Isothermal titration calorimetry analysis of the binding of L-malic acid to PA2652-LBD in the absence and presence of 2 or 20 mM of the antagonists, citraconic and D,L-methylsuccinic acids. Upper panel: Titration raw data for the injection of 9.6 μl aliquots of 1 mM of L-malic acid into 20 μM of protein in the absence and presence of antagonists (present both in the injector syringe and sample cell). Lower panel: Integrated, dilution heat corrected and concentration normalized peak areas fitted with the “One binding site” model of ORIGIN. The apparent binding constants are listed in Supplementary Table S2.

The above results suggest that chemoattractants (L-malic, D,L-bromosuccinic and L-citramalic acids) and antagonists (L-methylsuccinic and citraconic acids) may compete for binding to PA2652-LBD. In order to understand the mechanistic role of these antagonistics in the chemotaxis behaviour of *P. aeruginosa*, we first carried out ITC binding studies. In these assays, we measured the affinity of L-malic acid for PA2652-LBD in the presence of different concentrations of citraconic and D,L-methylsuccinic acids. Our data showed that heat released from the binding of L-malic acid was reduced as the antagonist concentration increased. The apparent affinity of L-malic acid decreased approximately by a factor of two in the presence of 2 mM antagonists and increasing the concentration of antagonists to 20 mM led to a further reduction in the apparent affinity for L-malic acid (Fig. 7 and Supplementary Table S2). Control assays titrating buffer or buffer/antagonist solutions with L-malic acid or L-malic acid/antagonist solutions resulted in small and uniform peaks indicative of dilution heats (Supplementary Fig. S8). Considering the close structural similarity of antagonists and L-malic acid (Fig. 3) it is likely that these compounds compete for binding at the same site at PA2652.

**Figure 8:**
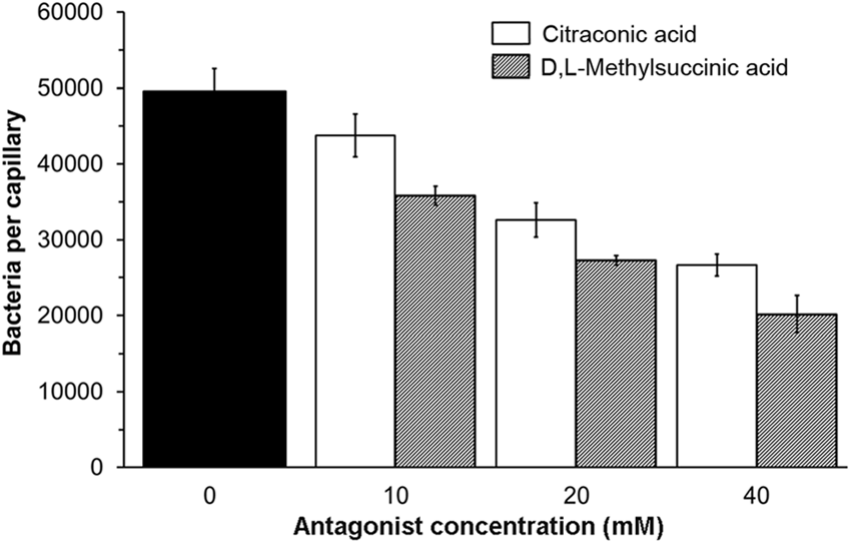
Antagonists reduce the magnitude of chemotaxis toward L-malic acid. Quantitative capillary chemotaxis assays of *P. aeruginosa* PAO1 toward 1 mM L-malic acid in the absence (black bars) and presence of different antagonist concentrations. Data are means and standard deviations from three biological replicates conducted in triplicate. Data were corrected with the number of cells that swam into buffer containing capillaries (4266 ± 1133).

Following the demonstration of competition *in vitro* we have assessed the influence of antagonists on the chemotaxis toward L-malic acid. To this end we conducted chemotaxis assays toward L-malic acid in the absence or presence of 10, 20 and 40 mM of citraconic and D,L-methylsuccinic acids (that were added to both, the bacterial suspension and the chemoattractant solution). Our results showed that antagonists reduced the chemotaxis toward L-malic acid, with higher decreases in the chemotactic response as the concentration of the antagonist was increased, therefore indicating that citraconic and methylsuccinic acids are inhibiting the activation of the chemotaxis signalling cascade triggered by L-malic acid (Fig. 8). To verify whether the presence of antagonists may have a global inhibitory effect on the chemotactic properties of *P. aeruginosa* we performed assays toward L-alanine, a chemoattractant recognized by the receptors PctA and PctB^15^, in the presence antagonists. As shown in Supplementary Fig. S9, the presence of 20 or 40 mM citraconic and D,L-methylsuccinic acids did not significantly alter the chemotaxis toward 1 mM L-alanine.

### The PA2652 chemoreceptor signals through the *che* chemosensory pathway

As mentioned in the introduction, *P. aeruginosa* has two chemosensory pathways that were found to be involved in chemotaxis, namely the *che* and *che2* pathways^9,10^. A recent bioinformatic study has predicted that PA2652 signals through the *che* pathway^44^. To verify this prediction we conducted chemotaxis assays to L-malic acid using the wt strain as well as mutants in *cheA1* and *cheA2*, encoding respectively the histidine kinases of the *che* and *che2* pathways. As shown in Supplementary Fig. S10, mutation of *cheA1* abolished chemotaxis to L-malic acid, whereas the response of the *cheA2* mutant was almost identical to that of wt strain, confirming bioinformatic predictions^44^.

### The effect of PA2652 in plant root colonization

*P. aeruginosa* is an ubiquitous pathogen and also able to colonize and infect different plants^20,45,46^. Previous studies using a number of different bacterial species have shown that chemotaxis to root exudates is an important prerequisite for efficient plant root colonization^47^. Taken together data from different plant species, malate and citrate are considered the most abundant organic acids in plant tissues and root exudates, and they can represent up to 25% of total photosynthate exuded by the plant^48,49^. Therefore, considering the important concentrations of malate in root exudates and using maize as model plants, we carried out competitive root colonization assays of the kanamycin resistant *P. aeruginosa* strain PAO1-Km and a mutant defective in *PA2652*. The assays showed that the fitness of the mutant in *PA2652* was comparable to that of the wild type strain (Supplementary Fig. S11).

## Discussion

Sensing of environmental signals by one- and two-component systems as well as chemotaxis signalling pathways occurs through sensor domains. Remarkably, the development of next generation sequencing technologies has allowed to determine that these three transduction mechanisms share a significant number of sensor domain types^4,50^. Additionally, the combination of computational and experimental approaches enabled the identification of inhibiting compounds, known as antagonists, that are sensed by one- and two-component systems. These molecules compete for binding to the sensor domains and block the agonist-induced response^51–54^. Importantly, there is increasing evidence indicating that signal antagonists can also act on chemoreceptors. Thus, it was shown that the binding of several compounds to the 4HB type LBDs of the Tar and MCP2201 chemoreceptors, from *E. coli* and *Comamonas testosteroni* respectively, did not cause any chemotactic responses^55–57^.

In this study we identified four new ligands for PA2652, which all occur naturally^58–61^. However, significant chemotaxis was only observed for two of them, bromosuccinic and citramalic acids - to our knowledge the first report of bacterial chemotaxis to both of these compounds. In contrast, no or very minor responses were observed for citraconic and methylsuccinic acids, respectively (Fig. 5). Additional *in vitro* experimentation showed that citraconic and methylsuccinic acids bind to the sCACHE domain of PA2652 and act as antagonists by competing for binding with chemoattractants (Fig. 7). Consequently, taxis of *P. aeruginosa* toward chemoattractants was reduced in the presence of these two antagonists (Fig. 8), as observed previously for other chemoreceptor antagonists^55,56^. Interestingly, malate was shown to be an antagonist of MCP2201 and its binding to the LBD of this chemoreceptor reduced the chemotactic behavior of *C. testosteroni* toward diverse aromatic compounds^56^. Taken together, our data illustrate that the action of antagonists is not only restricted to 4HB LBDs, but also occurs at chemoreceptors with a different LBD type. Could these findings be of applied interest? The increasing emergence of multidrug-resistant bacteria is challenging human health and new targets for the development of antibiotics are urgently needed^62^. Chemotaxis has been shown to be required for the full virulence of multiple human pathogens^63^ and drugs targeting chemosensory signalling pathways constitute promising approaches for the discovery of new antibiotics^64^. Since many drugs are based on antagonists^65,66^, the identification of compounds interfering with chemotactic transduction pathways may potentially enable the rational design of drugs inhibiting chemotaxis-associated processes.

CACHE domains are the most abundant sensor domains in chemoreceptors and sensor kinases^34,35^. Whereas *P. aeruginosa* PAO1 and *P. putida* KT2440 have an elevated number of dCACHE containing chemoreceptors, both strains have a single sCACHE domain containing chemoreceptor. Although both receptors bind organic acids, their ligand profiles are different. Whereas McpP binds several C2- and C3-carboxylic acids^36^, we show here that PA2652 binds several C2-substituted C4-dicarboxylic acids. Interestingly, the homologous chemoreceptor in the plant pathogen *P. syringae* pv. *actinidiae* was found to have a ligand profile that is very similar to that of *P. putida* KT2440^67^.

Several previous studies have shown that the affinity of ligands for the LBD correlate with the magnitude of the chemosensory output^16,43^. However, this correlation was not observed for the PA2652 ligands (Figs 3 and 5). In the initial study of PA2652, chemotaxis assays were performed using malate samples containing both isomers^33^. Here we show that PA2652 binds exclusively the L- but not the D-isomer of malic, citramalic and methylsuccinic acids (Fig. 2a and Supplementary Fig. S4). In this respect, clear parallels exist to McpP that binds only L-lactate but not D-lactate^36^. Many bacteria are able to synthesize D-malate^68,69^ and *Pseudomonas* species were found to metabolize both isomers^70^. However, L-malate is a common carboxylic acid whereas its D-isomer is less frequent^71^. In the context of sensory mechanisms for the regulation of the metabolism of organic acids, it has been proposed that common carboxylic acids are sensed in the periplasm whereas uncommon acids are sensed in the cytosol^69,71^. For example the DcuS sensor kinase, comprising a periplasmic LBD, senses L-malate^72^ whereas the cytosolic transcriptional regulator DmlR senses D-malate^73^. This differentiation appears to also apply to L-malate chemoreceptors. Chemoreceptors can sense their ligands either in the cytosol or the extracytoplasmic space^74^. A significant number of malate responsive chemoreceptors have been identified in a variety of different species and the corresponding information is summarized in Table 1. Whereas in some studies mixtures of D- and L-malate were used, other reports study the individual malate isomers. Interestingly, next to PA2652, the McpS receptor of *P. putida* KT2440^26^, McpM of *Ralstonia pseudosolanacearum*^75^ as well as the Pfl01_0728 and Pfl01_3768 of *P. fluorescens*^76^ were found to mediate specifically L-malate chemotaxis. All these receptors are predicted to possess a LBD in the periplasmic space confirming the hypothesis of extracytoplasmic sensing of common organic acids. The response of *R. pseudosolanacearum* to D-malate has also been investigated and was attributed to an fortuitous response of L-malate sensing receptors^77^. In contrast to other malate specific receptors, the LBD of both malate chemoreceptors of *R. pseudosolanacearum* have a 4HB domain (Table 1).

**Table 1:** Summary of information available on malate responsive chemoreceptors.

Name	Species	Ligands	Binding mode	LBD type^a^	Predicted LBD location^b^	Reference
PA2652	*P. aeruginosa* PAO1	L-malic, citramalic, citraconic, bromosuccinic and methylsuccinic acids	direct	sCACHE	periplasm	This work
McpS (PP4658)	*P. putida* KT2440	L-malic, oxalacetic, citric, isocitric, succinic, fumaric and butyric acids	direct	HBM	periplasm	^25,26^
McpM (GenBank accession no. LC005239)	*Ralstonia pseudosolanacearum* Ps29	L-malic, D-malic, D-tartaric, succinic, and fumaric acids	unknown	4HB	periplasm	^75,77^
McpT (GenBank accession no. LC005228)	*Ralstonia pseudosolanacearum* Ps29	D-malic acid, D-tartaric and L-tartaric acids	unknown	4HB	periplasm	^77^
McfS (Pput_4520)	*P. putida* F1	succinic, malic, citric and fumaric acids	unknown	HBM	periplasm	^29^
McfR (Pput_0339)	*P. putida* F1	succinic, malic and fumaric acids	unknown	4HBB	periplasm	^29^
McpS (Pfl01_0728)	*P. fluorescens* Pf0-1	L-malic and succinic acids	unknown	HBM	periplasm	^76^
McpT (Pfl01_3768)	*P. fluorescens* Pf0-1	L-malic and succinic acids	unknown	sCACHE	periplasm	^76^
CcmL (Tlp3)	*Campylobacter jejuni* 11168-O	chemoattractants: malic and fumaric acids, Ile, purine; chemorepellents: Lys, Arg, glucosamine, succinic acid, thiamine	direct	dCACHE	periplasm	^85^
MCP2201 (CtCNB1_2201)	*Comamonas testosteroni* CNB-1	malic acid (inhibitor of taxis to other organic acids), oxaloacetic, citric, isocitric, α-ketoglutaric, succinic and fumaric acids	direct	4HB	periplasm	^56^

^a^Based on the Pfam database^86^

^b^Based o the prediction of TM region using the DAS algorithm^39^.

The inspection of information available on the different malate responsive chemoreceptors (Table 1) also shows that these receptors differ in their LBD type. In general, chemoreceptor LBDs can be classified according to their size into clusters I and II^78^. Interestingly, malate responsive receptors include cluster I (sCACHE, 4HB) as well as cluster II (dCACHE, HBM) LBDs, and direct malate binding has been observed for all 4 LBD types (Table 1). This diversity in the molecular architecture of malate responsive receptors underlines the important physiological relevance of this ligand. Thus, as an example, the importance of malate chemotaxis has been reflected in *R. pseudosolanacearum* since a mutant defective in *mcpM* exhibits reduced virulence as compared to the wt strain in tomato plants^75^. Additionally, taxis to organic acids has been shown to be important for the colonization of the gastrointestinal tract of chicken by *Campylobacter jejuni*^79^.

Based on the hypothesis that ligand induced chemoreceptor dimerization is a prerequisite for signalling^41^, the effect of ligands on the oligomeric state of different LBD types has been investigated in the past^15,19,25,28,42,43^. The individual 4HB domains of receptors Tar, CtpH and PcaY_PP^19,42,43^ and HBM LBDs (receptors McpS and McpQ)^25,28^ were found to be largely monomeric in their ligand free state whereas in all cases the binding of the ligand induced complete LBD dimerization. This is due to the fact that ligands bind at the dimer interface and that amino acids from both monomers of the dimer establish contacts with the bound ligand^26,80^. In marked contrast, dCACHE LBDs of the PctA and PctB chemoreceptors were entirely monomeric in the absence and presence of ligands. Importantly, no information was available on the oligomeric state of sCACHE domains and we show here that yet another scenario applies to PA2652-LBD. Thus, in the absence of ligand, PA2652-LBD was entirely dimeric over the concentration range tested and L-malic acid did not have any effect on its oligomeric state (Fig. 4).

*P. aeruginosa* has two chemosensory pathways involved in chemotaxis. Chemotaxis to L-malic acid in the *cheA2* mutant strain was indistinguishable from that of the wt, whereas no response was observed in a mutant defective in *cheA1*. These results confirm the bioinformatic predictions^44^ but also demonstrate the exclusivity of the *che* pathway in mediating L-malic acid responses.

## Methods

### Bacterial Strains, Culture Media, and Growth Conditions

Bacterial strains used in this study are listed in Supplementary Table S3. *Pseudomonas aeruginosa* PAO1 and its derivative strains were routinely grown at 37 °C in Luria Broth (LB; 5 g yeast extract l^−1^, 10 g Bacto tryptone l^−1^ and 5 g NaCl l^−1^) or M9 medium supplemented with 1 mM MgSO4, 6 mg l^−1^ Fe-citrate, trace elements^81^ and 15 mM glucose as carbon source. For growth experiments to assess the capacity of PA2652-LBD ligands to support growth a sole C-source, PAO1 cells were pre-cultured overnight in M9 medium supplemented with 10 mM glucose and washed twice with M9 medium salts, prior to the inoculation of M9 medium containing10 mM of the different carbon sources. When necessary, the pH of the medium was adjusted to 7.0 prior to inoculation. Bacterial growth over the time was monitored using Bioscreen Microbiological Growth Analyser (Oy Growth Curves Ab Ltd, Helsinki, Finland). *Escherichia coli* strains were grown at 37 °C in LB. *Escherichia coli* DH5α was used as a host for gene cloning. When appropriate, antibiotics were used at the following final concentrations (in µg ml^−1^): kanamycin, 25 (*E. coli* strains) and 100 (*Pseudomonas* strains); tetracycline, 40.

### Construction of expression plasmid for PA2652-LBD

The DNA fragment encoding the LBD of PA2652 (Lys^34^–Thr^205^) was amplified from genomic DNA of *P. aeruginosa* PAO1 using primers 5′-TAATCATATGAAAAAACAGGCTGATGCCGA-3′ and 5′-TAATGTCGACTCAGGTGCCGATACGCTCGTC-3′ containing restriction sites for NdeI and SalI, respectively (underlined). The resulting PCR product was digested with NdeI and SalI and cloned into pET28b(+) using the same enzymes. The resulting plasmid, termed pET28b-PA2652LBD, was verified by DNA sequencing of the insert and flanking regions.

### Overexpression and purification of PA2652-LBD

*Escherichia coli* BL21 (DE3) containing pET28b-PA2652LBD was grown in 2 L Erlenmeyer flasks containing 400 ml LB medium supplemented with 50 μg ml^−1^ kanamycin at 30 °C. Once the culture reached an OD_600_ of 0.6, protein overexpression was induced by adding isopropyl β-D-1-thiogalactopyranoside (IPTG) to a concentration of 0.1 mM. Growth was continued at 16 °C overnight prior to cell harvest by centrifugation at 10 000 × *g* for 30 min at 4 °C. Cell pellets were resuspended in buffer A (20 mM Tris/HCl, 0.1 mM EDTA, 300 mM NaCl, 10 mM imidazole, 5% (v/v) glycerol, pH 8.0) and broken by French press treatment at 1000 psi. After centrifugation at 20 000 × *g* for 1 h, the supernatant was loaded onto a 5 ml HisTrap column (Amersham Bioscience), previously equilibrated with five column volumes of buffer A, washed with buffer A containing 35 mM of imidazole and eluted with a 35–300 mM imidazole gradient in buffer A. Protein-containing fractions were pooled and dialyzed for immediate analysis.

### Thermal Shift Assay based high-throughput ligand screening

Thermal shift assays were performed on a MyIQ2 Real-Time PCR instrument (BioRad). Ligands from the different compound arrays (Biolog, Hayward, CA, USA; see Supplementary Fig. S3) were dissolved in 50 μl of MilliQ water, which, according to the manufacturer, corresponds to a concentration of 10–20 mM. Screening was performed using 96 wells plates. Each well contained 20 μM of protein dialyzed into TNG buffer (20 mM Tris/HCl, 150 mM NaCl, 10% (v/v) glycerol, pH 6.7), 2.5 μl of the resuspended compounds and SYPRO Orange (Life Technologies) at 5× concentration. In a single well (ligand free protein) the compound was substituted by water. Samples were heated from 23 °C to 85 °C at a scan rate of 1 °C/min. The protein unfolding curves were obtained by following the changes in SYPRO Orange fluorescence. Melting temperatures were determined using the first derivative values from the raw fluorescence data.

### Isothermal titration calorimetry binding studies

Experiments were conducted on a VP-microcalorimeter (Microcal, Amherst, MA, USA) at 20 °C. PA2652-LBD was dialyzed overnight against TNG buffer, adjusted to a concentration of 20–35 μM and placed into the sample cell of the instrument. The protein was titrated by the injection of 9.6–14.4 μl aliquots of 1–20 mM ligand solutions that were prepared in TNG buffer (20 mM Tris/HCl, 150 mM NaCl, 10% (v/v) glycerol, pH 6.8) immediately before use. The mean enthalpies measured from the injection of ligands into buffer were subtracted from raw titration data prior to data analysis with the MicroCal version of ORIGIN. Data were fitted with the “One binding site model”.

### Analytical ultracentrifugation studies

Experiments were performed on a Beckman Coulter Optima XL-A analytical ultracentrifuge (Beckman-Coulter, Palo Alto, CA, USA) equipped with UV-visible absorbance detection system, using an An50Ti 8-hole rotor and 12 mm path-length charcoal-filled epon double-sector centrepieces. The experiments were carried out at a rotor speed of 48 000 rpm and 7 °C using 400 µL samples of proteins dialyzed into in PIPES buffer (20 mM PIPES, pH 7.0). Protein was at 5–20 μM and L-malic acid (stock solution made up in dialysis buffer) was added at a final concentration of 1 mM. Dialysis buffer with and without ligand were used as reference. Light at a wavelength of 234 nm was recorded in the absorbance optics mode. A least squares boundary modelling of the data was used to calculate sedimentation coefficient distributions with the size-distribution c(s) method implemented in the SEDFIT v11.71 software^82^. The Svedberg equation allowed us to estimate the experimental molecular weight from the sedimentation and diffusion coefficients obtained. Buffer density (*ρ* = 1.0015 g/mL) and viscosity (*η* = 0.01449 Poise) at 7 °C were calculated from the buffer composition using SEDNTERP software^83^. This software was also used to calculate the partial specific volume (0.721 ml/g) and the molecular weight (21.5 kDa) of PA2652-LBD from its sequence.

### Plasmid construction for genetic complementation assays

For the construction of the complementing plasmid, a full copy of the *PA2652* gene was amplified by PCR using the primers PA2652-NdeI-F (5′-TAATCATATGATGCGTCTGACCCTGAAATCC-3′) and PA2652-BamHI-R (5′- TAATGGATCCGACAGGAAGGCTCTGTGGCG-3′). Restriction sites for NdeI and BamHI are underlined. The resulting fragment was digested with NdeI and BamHI and cloned into the same sites in pBBR1MCS2_START to generate pBBR2652f4. The insert was confirmed by PCR and sequencing, and pBBR2652f4 was used to transform the *PA2652* defective mutant by electroporation.

### Quantitative Capillary Chemotaxis Assays

Overnight cultures of *P. aeruginosa* strains were diluted to an OD_660_ of 0.05 in MS medium (30 mM Na_2_HPO_4_, 20 mM KH_2_PO_4_, 25 mM NH_4_NO_3_, 1 mM MgSO_4_) supplemented with 6 mg l^−1^ Fe-citrate, trace elements^81^ and 15 mM glucose as carbon source, and grown at 37 °C with orbital shaking (200 rpm). At an OD_660_ of 0.4 (early stationary phase of growth) cultures were centrifuged at 1,700 × *g* for 5 min and the resulting pellet was washed twice with chemotaxis buffer (50 mM potassium phosphate, 20 mM EDTA, 0.05% (v/v) glycerol, pH 7.0). Subsequently, the cells were resuspended in the same buffer, adjusted to an OD_660_ of 0.1 and 230 µl aliquots of the bacterial cultures were placed into 96-well plates. For the quantitative assays, one-microliter capillary tubes (Microcaps, Drummond Scientific, Ref. P1424) were heat-sealed at one end and filled with either the chemotaxis buffer (negative control) or chemotaxis buffer containing the chemoeffectors to test. The capillaries were immersed into the bacterial suspensions at its open end. After 30 min at room temperature, capillaries were removed from the bacterial suspensions, rinsed with sterile water and the content expelled into 1 ml of M9 medium salts. Serial dilutions were plated onto M9 minimal medium (containing the appropriate antibiotics) supplemented with 15 mM glucose as carbon source. The number of colony forming units was determined after overnight incubation. In all cases, data were corrected with the number of cells that swam into buffer containing capillaries.

To determine the effect of antagonists in the chemotactic properties of PAO1, the assay was performed as previously described with two minor modifications: (i) Capillary tubes were filled with chemotaxis buffer containing 1 mM of the chemoattractants (L-malic acid or L-alanine) and 10–40 mM of the antagonists (methylsuccinic or citraconic acids); (ii) Bacterial cultures were washed with chemotaxis buffer and cells were finally resuspended in the same buffer but containing equimolar concentrations of methylsuccinic/citraconic acids to those present in the capillary tubes.

### Competitive Root Colonization Assays

Sterilization and germination of maize seeds was carried out as described previously^84^. Subsequently, 10 mL of M9 salts containing a 10^6^ CFU/ml 1:1 mixture of PAO1-Km (wild type) and PAO-PA2652 (mutant) were added to 50 ml Sterilin tubes containing 40 g of sterile washed silica sand. Thereafter, one sterile seed was planted per Sterilin tube containing the inoculated silica sand. Plants were maintained at 24 °C with a daily light period of 16 h. After 6 days, bacterial cells were recovered from the rhizosphere and serial dilutions were plated on LB-agar medium supplemented with kanamycin or tetracycline to select PAO1-Km or the *PA2652* mutant strain, respectively.

## Electronic supplementary material

Supplementary material
