# Supplementary material for: The activity of the C4-dicarboxylic acid chemoreceptor of Pseudomonas aeruginosa is controlled by chemoattractants and antagonists
Source: Sci Rep. 2018 Feb 1;8:2102. doi: 10.1038/s41598-018-20283-7 (PMC5795001; doi:10.1038/s41598-018-20283-7)
Supplement: Supplementary file 1 — Supplementary material [file 41598_2018_20283_MOESM1_ESM.pdf]

Supplementary material

to

The activity of the C4-dicarboxylic acid chemoreceptor of *Pseudomonas aeruginosa* is controlled by chemoattractants and antagonists

David Martín-Mora[a], Álvaro Ortega[a], Francisco J. Pérez-Maldonado[a], Tino Krell[a], Miguel A. Matilla[a,]*

[a]Department of Environmental Protection, Estación Experimental del Zaidín, Consejo Superior de Investigaciones Científicas, Granada, Spain.

*Address correspondence to Miguel A. Matilla, Estación Experimental del Zaidín, Consejo Superior de Investigaciones Científicas, Prof. Albareda 1, 18008 Granada, Spain; Phone: + 34 958 181600, Fax: + 34 958 135740, e-mail: miguel.matilla@eez.csic.es.

```
                       10        20        30        40        50        60
                        |         |         |         |         |         |
McpP           MNTLRSMSISRRLWLILVVAVAMLVVLGLLMLRQIHGD---------LYQAKAEKTRHVV
PA2652         MMRLTLKSKVLLLAMVPVLLFALVLSGGAVLILKKQADAEVKDTRERLLGDRRAELEHYV

                       70        80        90       100       110       120
                        |         |         |         |         |         |
McpP           QTAAGVLAYYQGLEAAGTLSREAAQQQALQVVRALRYDHDDYFWINDLGPKMIMHPANPK
PA2652         QIAMGSIQAEYDRSANGDLN---ARAEAIARLSKIKYGKDGYIFGYDSQVVRLFRGDSPV

                      130       140       150       160       170       180
                        |         |         |         |         |         |
McpP           LDDQDLSAIRDPDGFAVFNEMVALARQQDAGPVNYRWPKPG-ASEPVAKTSYIQLFKPWG
PA2652         DVGKSFRDRRDPSGVYLNRELVEAGRN-GSHYVTYTSPLPGNESVMVPKLSYTLYLPKWD

                      190       200       210       220       230       240
                        |         |         |         |         |         |
McpP           WIIGSGVYVDDVQAEFARQLRD---------ASLVGVGIALLMALVVM--LIARSIARPL
PA2652         MVIGSAINLDGVEAQLVEIKQDIDERIGTLIASIVGIAGVLLVVLLVIGLAVANAMLRPL

                      250       260       270       280       290       300
                        |         |         |         |         |         |
McpP           QEAVQAMGNIASGESDLTRRLDTHGSDEITHLGEHFNRFNGKLQGVVGQLQGAAHALAQS
PA2652         HQIRQNLDDIAAGEGDLTRRLPVTSYDELGELAGSFNRFVEKIHGLVRQIAGMTGDLKQL

                      310       320       330       340       350       360
                        |         |         |         |         |         |
McpP           AGHVGDNAGAAQQRSAQQSLQMDQVATAVNEVTYAVQDVAKTAEQAAGEMRTAQQQVTHG
PA2652         VEQMSAQAERSEQAMERQRHETDQVATAINEMSAAAHEVAQSAQRAAEAAQQTDHEGQAA

                      370       380       390       400       410       420
                        |         |         |         |         |         |
McpP           QQAIHGSLAQIDRLSLTIDEAVQVIRDLAGHSTRIGGVLDVIRSIAEQTNLLALNAAIEA
PA2652         KRVVDGSIERIHALVDEIRDSGTSLDSLQQDVQSIVSVLGVIRSIAEQTNLLALNAAIEA

                      430       440       450       460       470       480
                        |         |         |         |         |         |
McpP           ARAGEQGRGFAVVADEVRLLAQRTAQSTAEIHTMIEHLQSQSDAAVKAIDTSSEASRQTV
PA2652         ARAGEAGRGFAVVADEVRALASRTQQSTQEIQGMIDRLQQGTNAAVDAMRRSGEAGEGTS

                      490       500       510       520       530       540
                        |         |         |         |         |         |
McpP           EQAREAGASLDAINQVLNNLTALNASIASATLQQSHVVEEINRNVLDTAGLSQQTADAAR
PA2652         NQANQAGDSLDAIAQLIATINAMNAQIASAAEEQTAVAEEINRSVHQIAGAVDSVADEAQ

                      550       560
                        |         |
McpP           QSSDAGVALGRLSEELEQLLRQFRV
PA2652         QGAQTARSLAQLGQGLGRLVGQFRI
```

Supplementary Figure S1. Sequence alignment of sCACHE LBD containing chemoreceptors in *P. putida* KT2440 (McpP) and *P. aeruginosa* PAO1 (PA2652). The alignment was performed in the slow mode using the CLUSTALW multiple alignment tool[1] of the NPS@ suite[2]. The GONNET protein weight matrix was used and gap opening and gap extension penalties of 10 and 0.1 were applied, respectively.

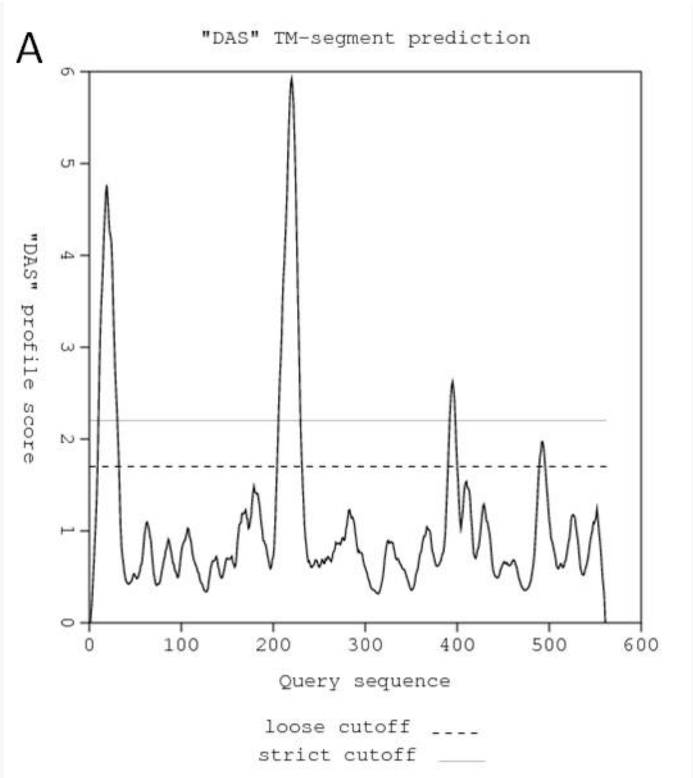

B

MMRLTLKSKVLLLAMVPVLLFALVLSGGAVLIL**KKQADAEVKDTRERLLGDRRAELEHYVQIAM**
GSIQAEYDRSANGDLNARAEAIARLSKIKYGKDGYIFGYDSQVVRLFRGDSPVDVGKSFRDRRD
PSGVYLNRELVEAGRNGSHYVTYTSPLPGNESVMVPKLSYTLYLPKWDMVIGSAINLDGVEAQ
LVEIKQDIDERIGTLIASIVGIAGVLLVVLLVIGLAVANAMLRPLHQIRQNLDDIAAGEGDLTRRLPV
TSYDELGELAGSFNRFVEKIHGLVRQIAGMTGDLKQLVEQMSAQAERSEQAMERQRHETDQVATAI
NEMSAAAHEVAQSAQRAAEAAQQTDHEGQAAKRVVDGSIERIHALVDEIRDSGTSLDSLQQDVQSI
VSVLGVIRSIAEQTNLLALNAAIEAARAGEAGRGFAVVADEVRALASRTQQSTQEIQGMIDRLQQGT
NAAVDAMRRSGEAGEGTSNQANQAGDSLDAIAQLIATINAMNAQIASAAEEQTAVAEEINRSVHQIA
GAVDSVADEAQQGAQTARSLAQLGQGLGRLVGQFRI

Supplementary Figure S2. Prediction of transmembrane regions in PA2652 using the DAS algorithm. A, Graphical output from DAS[3]. **B**, Sequence of PA2652 showing in red the transmembrane regions and the LBD sequence cloned into the expression plasmid in blue.

PM1 array

A1<br>Negative Control	A2<br>L-Arabinose	A3<br>N-Acetyl-D-Glucosamine	A4<br>D-Saccharic Acid	A5<br>Succinic Acid	A6<br>D-Galactose	A7<br>L-Aspartic Acid	A8<br>L-Proline	A9<br>D-Alanine	A10<br>D-Trehalose	A11<br>D-Mannose	A12<br>Dulcitol
B1<br>D-Serine	B2<br>D-Sorbitol	B3<br>Glycerol	B4<br>L-Fucose	B5<br>D-Glucuronic Acid	B6<br>D-Gluconic Acid	B7<br>D,L-α-Glycerol-Phosphate	B8<br>D-Xylose	B9<br>L-Lactic Acid	B10<br>Formic Acid	B11<br>D-Mannitol	B12<br>L-Glutamic Acid
C1<br>D-Glucose-6-Phosphate	C2<br>D-Galactonic Acid-γ-Lactone	C3<br>D,L-Malic Acid	C4<br>D-Ribose	C5<br>Tween 20	C6<br>L-Rhamnose	C7<br>D-Fructose	C8<br>Acetic Acid	C9<br>α-D-Glucose	C10<br>Maltose	C11<br>D-Melibiose	C12<br>Thymidine
D-1<br>L-Asparagine	D2<br>D-Aspartic Acid	D3<br>D-Glucosaminic Acid	D4<br>1,2-Propanediol	D5<br>Tween 40	D6<br>α-Keto-Glutaric Acid	D7<br>α-Keto-Butyric Acid	D8<br>α-Methyl-D-Galactoside	D9<br>α-D-Lactose	D10<br>Lactulose	D11<br>Sucrose	D12<br>Uridine
E1<br>L-Glutamine	E2<br>m-Tartaric Acid	E3<br>D-Glucose-1-Phosphate	E4<br>D-Fructose-6-Phosphate	E5<br>Tween 80	E6<br>α-Hydroxy Glutaric Acid-γ-Lactone	E7<br>α-Hydroxy Butyric Acid	E8<br>β-Methyl-D-Glucoside	E9<br>Adonitol	E10<br>Maltotriose	E11<br>2-Deoxy Adenosine	E12<br>Adenosine
F1<br>Glycyl-L-Aspartic Acid	F2<br>Citric Acid	F3<br>m-Inositol	F4<br>D-Threonine	F5<br>Fumaric Acid	F6<br>Bromo Succinic Acid	F7<br>Propionic Acid	F8<br>Mucic Acid	F9<br>Glycolic Acid	F10<br>Glyoxylic Acid	F11<br>D-Cellobiose	F12<br>Inosine
G1<br>Glycyl-L-Glutamic Acid	G2<br>Tricarballylic Acid	G3<br>L-Serine	G4<br>L-Threonine	G5<br>L-Alanine	G6<br>L-Alanyl-Glycine	G7<br>Acetoacetic Acid	G8<br>N-Acetyl-β-D-Mannosamine	G9<br>Mono Methyl Succinate	G10<br>Methyl Pyruvate	G11<br>D-Malic Acid	G12<br>L-Malic Acid
H1<br>Glycyl-L-Proline	H2<br>p-Hydroxy Phenyl Acetic Acid	H3<br>m-Hydroxy Phenyl Acetic Acid	H4<br>Tyramine	H5<br>D-Psicose	H6<br>L-Lyxose	H7<br>Glucuronamide	H8<br>Pyruvic Acid	H9<br>L-Galactonic Acid-γ-Lactone	H10<br>D-Galacturonic Acid	H11<br>Phenylethyl-amine	H12<br>2-Aminoethanol

PM2A array

A1<br>Negative Control	A2<br>Chondroitin Sulfate C	A3<br>α-Cyclodextrin	A4<br>β-Cyclodextrin	A5<br>γ-Cyclodextrin	A6<br>Dextrin	A7<br>Gelatin	A8<br>Glycogen	A9<br>Inulin	A10<br>Laminarin	A11<br>Mannan	A12<br>Pectin
B1<br>N-Acetyl-D-Galactosamine	B2<br>N-Acetyl-Neuraminic Acid	B3<br>β-D-Allose	B4<br>Amygdalin	B5<br>D-Arabinose	B6<br>D-Arabitol	B7<br>L-Arabitol	B8<br>Arbutin	B9<br>2-Deoxy-D-Ribose	B10<br>i-Erythritol	B11<br>D-Fucose	B12<br>3-0-β-D-Galacto-pyranosyl-D-Arabinose
C1<br>Gentiobiose	C2<br>L-Glucose	C3<br>Lactitol	C4<br>D-Melezitose	C5<br>Maltitol	C6<br>a-Methyl-D-Glucoside	C7<br>β-Methyl-D-Galactoside	C8<br>3-Methyl Glucose	C9<br>β-Methyl-D-Glucuronic Acid	C10<br>α-Methyl-D-Mannoside	C11<br>β-Methyl-D-Xyloside	C12<br>Palatinose
D1<br>D-Raffinose	D2<br>Salicin	D3<br>Sedoheptulosan	D4<br>L-Sorbose	D5<br>Stachyose	D6<br>D-Tagatose	D7<br>Turanose	D8<br>Xylitol	D9<br>N-Acetyl-D-Glucosaminitol	D10<br>γ-Amino Butyric Acid	D11<br>δ-Amino Valeric Acid	D12<br>Butyric Acid
E1<br>Capric Acid	E2<br>Caproic Acid	E3<br>Citraconic Acid	E4<br>Citramalic Acid	E5<br>D-Glucosamine	E6<br>2-Hydroxy Benzoic Acid	E7<br>4-Hydroxy Benzoic Acid	E8<br>β-Hydroxy Butyric Acid	E9<br>γ-Hydroxy Butyric Acid	E10<br>a-Keto-Valeric Acid	E11<br>Itaconic Acid	E12<br>5-Keto-D-Gluconic Acid
F1<br>D-Lactic Acid Methyl Ester	F2<br>Malonic Acid	F3<br>Melibionic Acid	F4<br>Oxalic Acid	F5<br>Oxalomalic Acid	F6<br>Quinic Acid	F7<br>D-Ribono-1,4-Lactone	F8<br>Sebacic Acid	F9<br>Sorbic Acid	F10<br>Succinamic Acid	F11<br>D-Tartaric Acid	F12<br>L-Tartaric Acid
G1<br>Acetamide	G2<br>L-Alaninamide	G3<br>N-Acetyl-L-Glutamic Acid	G4<br>L-Arginine	G5<br>Glycine	G6<br>L-Histidine	G7<br>L-Homoserine	G8<br>Hydroxy-L-Proline	G9<br>L-Isoleucine	G10<br>L-Leucine	G11<br>L-Lysine	G12<br>L-Methionine
H1<br>L-Ornithine	H2<br>L-Phenylalanine	H3<br>L-Pyroglutamic Acid	H4<br>L-Valine	H5<br>D,L-Carnitine	H6<br>Sec-Butylamine	H7<br>D.L-Octopamine	H8<br>Putrescine	H9<br>Dihydroxy Acetone	H10<br>2,3-Butanediol	H11<br>2,3-Butanone	H12<br>3-Hydroxy 2-Butanone

PM3B array

A1<br>Negative Control	A2<br>Ammonia	A3<br>Nitrite	A4<br>Nitrate	A5<br>Urea	A6<br>Biuret	A7<br>L-Alanine	A8<br>L-Arginine	A9<br>L-Asparagine	A10<br>L-Aspartic Acid	A11<br>L-Cysteine	A12<br>L-Glutamic Acid
B1<br>L-Glutamine	B2<br>Glycine	B3<br>L-Histidine	B4<br>L-Isoleucine	B5<br>L-Leucine	B6<br>L-Lysine	B7<br>L-Methionine	B8<br>L-Phenylalanine	B9<br>L-Proline	B10<br>L-Serine	B11<br>L-Threonine	B12<br>L-Tryptophan
C1<br>L-Tyrosine	C2<br>L-Valine	C3<br>D-Alanine	C4<br>D-Asparagine	C5<br>D-Aspartic Acid	C6<br>D-Glutamic Acid	C7<br>D-Lysine	C8<br>D-Serine	C9<br>D-Valine	C10<br>L-Citrulline	C11<br>L-Homoserine	C12<br>L-Ornithine
D-1<br>N-Acetyl-L-Glutamic Acid	D2<br>N-Phthaloyl-L-Glutamic Acid	D3<br>L-Pyroglutamic Acid	D4<br>Hydroxylamine	D5<br>Methylamine	D6<br>N-Amylamine	D7<br>N-Butylamine	D8<br>Ethylamine	D9<br>Ethanolamine	D10<br>Ethylenediamine	D11<br>Putrescine	D12<br>Agmatine
E1<br>Histamine	E2<br>β-Phenylethyl-amine	E3<br>Tyramine	E4<br>Acetamide	E5<br>Formamide	E6<br>Glucuronamide	E7<br>D,L-Lactamide	E8<br>D-Glucosamine	E9<br>D-Galactosamine	E10<br>D-Mannosamine	E11<br>N-Acetyl-D-Glucosamine	E12<br>N-Acetyl-D-Galactosamine
F1<br>N-Acetyl-D-Mannosamine	F2<br>Adenine	F3<br>Adenosine	F4<br>Cytidine	F5<br>Cytosine	F6<br>Guanine	F7<br>Guanosine	F8<br>Thymine	F9<br>Thymidine	F10<br>Uracil	F11<br>Uridine	F12<br>Inosine
G1<br>Xanthine	G2<br>Xanthosine	G3<br>Uric Acid	G4<br>Alloxan	G5<br>Allantoin	G6<br>Parabanic Acid	G7<br>D,L-α-Amino-N-Butyric Acid	G8<br>γ-Amino-N-Butyric Acid	G9<br>ε-Amino-N-Caproic Acid	G10<br>D,L-α-Amino-Caprylic Acid	G11<br>δ-Amino-N-Valeric Acid	G12<br>α-Amino-N-Valeric Acid
H1<br>Ala-Asp	H2<br>Ala-Gln	H3<br>Ala-Glu	H4<br>Ala-Gly	H5<br>Ala-His	H6<br>Ala-Leu	H7<br>Ala-Thr	H8<br>Gly-Asn	H9<br>Gly-Gln	H10<br>Gly-Glu	H11<br>Gly-Met	H12<br>Met-Ala

PM4A array

A1<br>Negative Control	A2<br>Phosphate	A3<br>Pyrophosphate	A4<br>Trimeta-phosphate	A5<br>Tripoly-phosphate	A6<br>Triethyl Phosphate	A7<br>Hypophosphite	A8<br>Adenosine- 2'-monophosphate	A9<br>Adenosine- 3'-monophosphate	A10<br>Adenosine- 5'-monophosphate	A11<br>Adenosine- 2',3'-cyclic monophosphate	A12<br>Adenosine- 3',5'-cyclic monophosphate
B1<br>Thiophosphate	B2<br>Dithiophosphate	B3<br>D,L-α-Glycerol Phosphate	B4<br>β-Glycerol Phosphate	B5<br>Carbamyl Phosphate	B6<br>D-2-Phospho-Glyceric Acid	B7<br>D-3-Phospho-Glyceric Acid	B8<br>Guanosine- 2'-monophosphate	B9<br>Guanosine- 3'-monophosphate	B10<br>Guanosine- 5'-monophosphate	B11<br>Guanosine- 2',3'-cyclic monophosphate	B12<br>Guanosine- 3',5'-cyclic monophosphate
C1<br>Phosphoenol Pyruvate	C2<br>Phospho-Glycolic Acid	C3<br>D-Glucose-1-Phosphate	C4<br>D-Glucose-6-Phosphate	C5<br>2-Deoxy-D-Glucose 6-Phosphate	C6<br>D-Glucosamine-6-Phosphate	C7<br>6-Phospho-Gluconic Acid	C8<br>Cytidine- 2'-monophosphate	C9<br>Cytidine- 3'-monophosphate	C10<br>Cytidine- 5'-monophosphate	C11<br>Cytidine- 2',3'-cyclic monophosphate	C12<br>Cytidine- 3',5'-cyclic monophosphate
D1<br>D-Mannose-1-Phosphate	D2<br>D-Mannose-6-Phosphate	D3<br>Cysteamine-S-Phosphate	D4<br>Phospho-L-Arginine	D5<br>O-Phospho-D-Serine	D6<br>O-Phospho-L-Serine	D7<br>O-Phospho-L-Threonine	D8<br>Uridine- 2'-monophosphate	D9<br>Uridine- 3'-monophosphate	D10<br>Uridine- 5'-monophosphate	D11<br>Uridine- 2',3'-cyclic monophosphate	D12<br>Uridine- 3',5'-cyclic monophosphate
E1<br>O-Phospho-D-Tyrosine	E2<br>O-Phospho-L-Tyrosine	E3<br>Phosphocreatine	E4<br>Phosphoryl Choline	E5<br>O-Phosphoryl-Ethanolamine	E6<br>Phosphono Acetic Acid	E7<br>2-Aminoethyl Phosphonic Acid	E8<br>Methylene Diphosphonic Acid	E9<br>Thymidine- 3'-monophosphate	E10<br>Thymidine- 5'-monophosphate	E11<br>Inositol Hexaphosphate	E12<br>Thymidine 3',5'-cyclic monophosphate
F1<br>Negative Control	F2<br>Sulfate	F3<br>Thiosulfate	F4<br>Tetrathionate	F5<br>Thiophosphate	F6<br>Dithiophosphate	F7<br>L-Cysteine	F8<br>D-Cysteine	F9<br>L-Cysteinyl-Glycine	F10<br>L-Cysteic Acid	F11<br>Cysteamine	F12<br>L-Cysteine Sulfinic Acid
G1<br>N-Acetyl-L-Cysteine	G2<br>S-Methyl-L-Cysteine	G3<br>Cystathionine	G4<br>Lanthionine	G5<br>Glutathione	G6<br>D,L-Ethionine	G7<br>L-Methionine	G8<br>D-Methionine	G9<br>Glycyl-L-Methionine	G10<br>N-Acetyl-D,L-Methionine	G11<br>L- Methionine Sulfoxide	G12<br>L-Methionine Sulfone
H1<br>L-Djenkolic Acid	H2<br>Thiourea	H3<br>1-Thio-β-D-Glucose	H4<br>D,L-Lipoamide	H5<br>Taurocholic Acid	H6<br>Taurine	H7<br>Hypotaurine	H8<br>p-Amino Benzene Sulfonic Acid	H9<br>Butane Sulfonic Acid	H10<br>2-Hydroxyethane Sulfonic Acid	H11<br>Methane Sulfonic Acid	H12<br>Tetramethylene Sulfone

PM5 array

A1<br>Negative Control	A2<br>Positive Control	A3<br>L-Alanine	A4<br>L-Arginine	A5<br>L-Asparagine	A6<br>L-Aspartic Acid	A7<br>L-Cysteine	A8<br>L-Glutamic Acid	A9<br>Adenosine-3',5'-cyclic monophosphate	A10<br>Adenine	A11<br>Adenosine	A12<br>2'-Deoxy Adenosine
B1<br>L-Glutamine	B2<br>Glycine	B3<br>L-Histidine	B4<br>L-Isoleucine	B5<br>L-Leucine	B6<br>L-Lysine	B7<br>L-Methionine	B8<br>L-Phenylalanine	B9<br>Guanosine-3',5'-cyclic monophosphate	B10<br>Guanine	B11<br>Guanosine	B12<br>2'-Deoxy Guanosine
C1<br>L-Proline	C2<br>L-Serine	C3<br>L-Threonine	C4<br>L-Tryptophan	C5<br>L-Tyrosine	C6<br>L-Valine	C7<br>L-isoleucine + L-Valine	C8<br>trans-4-Hydroxy L-Proline	C9<br>(5) 4-Amino-Imidazole-4(5)-Carboxamide	C10<br>Hypoxanthine	C11<br>Inosine	C12<br>2'-Deoxy Inosine
D1<br>L-Ornithine	D2<br>L-Citrulline	D3<br>Chorismic Acid	D4<br>(-)Shikimic Acid	D5<br>L-Homoserine Lactone	D6<br>D-Alanine	D7<br>D-Aspartic Acid	D8<br>D-Glutamic Acid	D9<br>D,L-α,ε-Diamino-pimelic Acid	D10<br>Cytosine	D11<br>Cytidine	D12<br>2'-Deoxy Cytidine
E1<br>Putrescine	E2<br>Spermidine	E3<br>Spermine	E4<br>Pyridoxine	E5<br>Pyridoxal	E6<br>Pyridoxamine	E7<br>β-Alanine	E8<br>D-Pantothenic Acid	E9<br>Orotic Acid	E10<br>Uracil	E11<br>Uridine	E12<br>2'-Deoxy Uridine
F1<br>Quinolinic Acid	F2<br>Nicotinic Acid	F3<br>Nicotinamide	F4<br>β-Nicotinamide Adenine Dinucleotide	F5<br>δ-Amino-Levulinic Acid	F6<br>Hematin	F7<br>Deferoxamine Mesylate	F8<br>D-(+)-Glucose	F9<br>N-Acetyl D-Glucosamine	F10<br>Thymine	F11<br>Glutathione (reduced form)	F12<br>Thymidine
G1<br>Oxaloacetic Acid	G2<br>D-Biotin	G3<br>Cyano-Cobalamine	G4<br>p-Amino-Benzoic Acid	G5<br>Folic Acid	G6<br>Inosine + Thiamine	G7<br>Thiamine	G8<br>Thiamine Pyrophosphate	G9<br>Riboflavin	G10<br>Pyrrolo-Quinoline Quinone	G11<br>Menadione	G12<br>m-Inositol
H1<br>Butyric Acid	H2<br>D,L-α-Hydroxy-Butyric Acid	H3<br>α-Keto-Butyric Acid	H4<br>Caprylic Acid	H5<br>D,L-α-Lipoic Acid (oxidized form)	H6<br>D,L-Mevalonic Acid	H7<br>D,L-Carnitine	H8<br>Choline	H9<br>Tween 20	H10<br>Tween 40	H11<br>Tween 60	H12<br>Tween 80

Supplementary Figure S3. Compounds used for Differential Fluorimetry based ligand screening of PA2652-LBD. Compounds were from 5 different compound arrays commercialised by Biolog (Hayward, CA, USA).

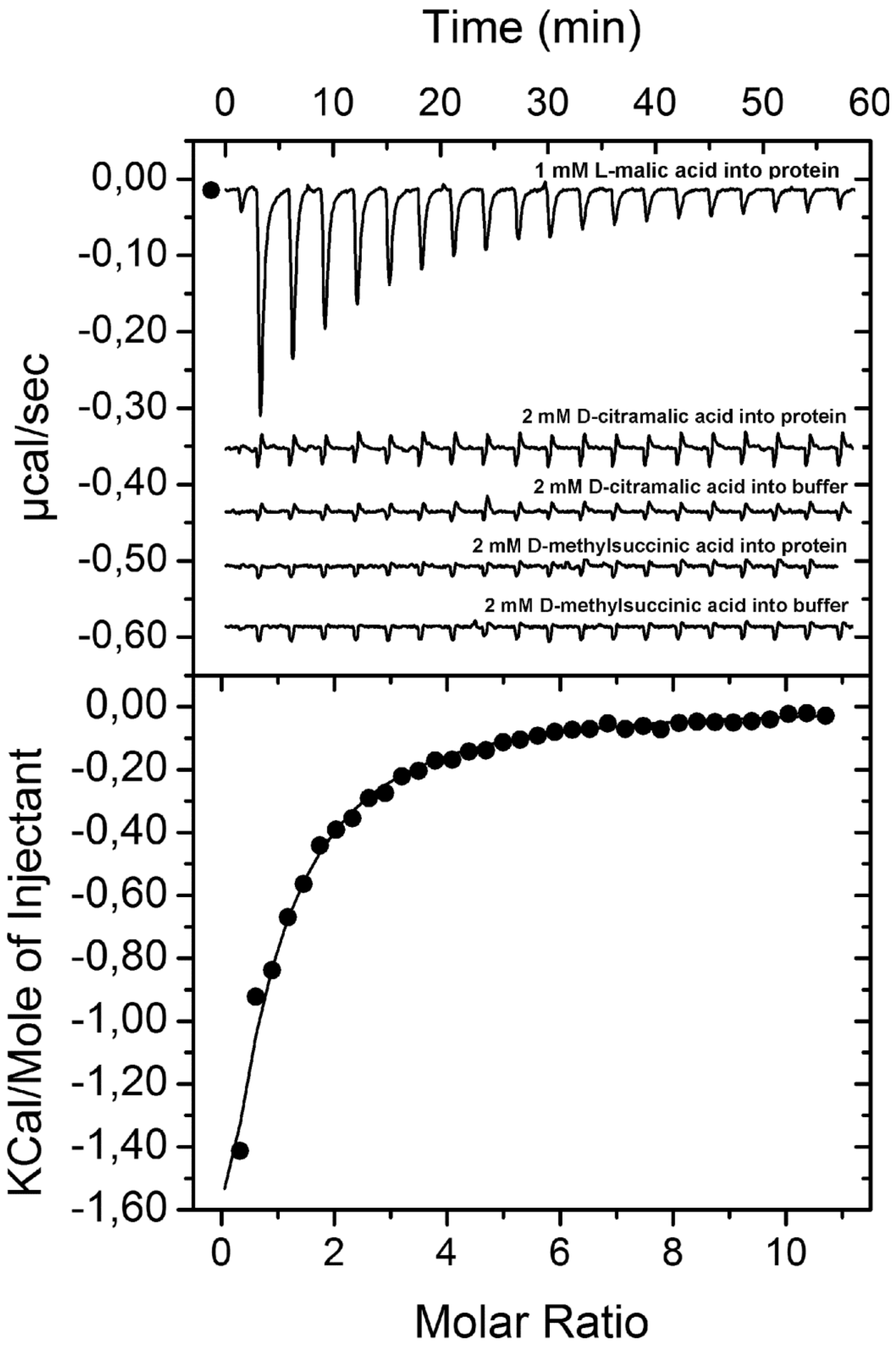

Supplementary Figure S4. Microcalorimetric titrations of L- and D-enantiomers to PA2652-LBD. The upper panels are the titration raw data for the injection of 8-11.2 µl aliquots of 1–2 mM ligand solutions into 20 µM of protein. The lower panels are the integrated, dilution heat corrected and concentration normalized peak areas fitted with the "One binding site" model of ORIGIN. L-malic acid was used as positive control.

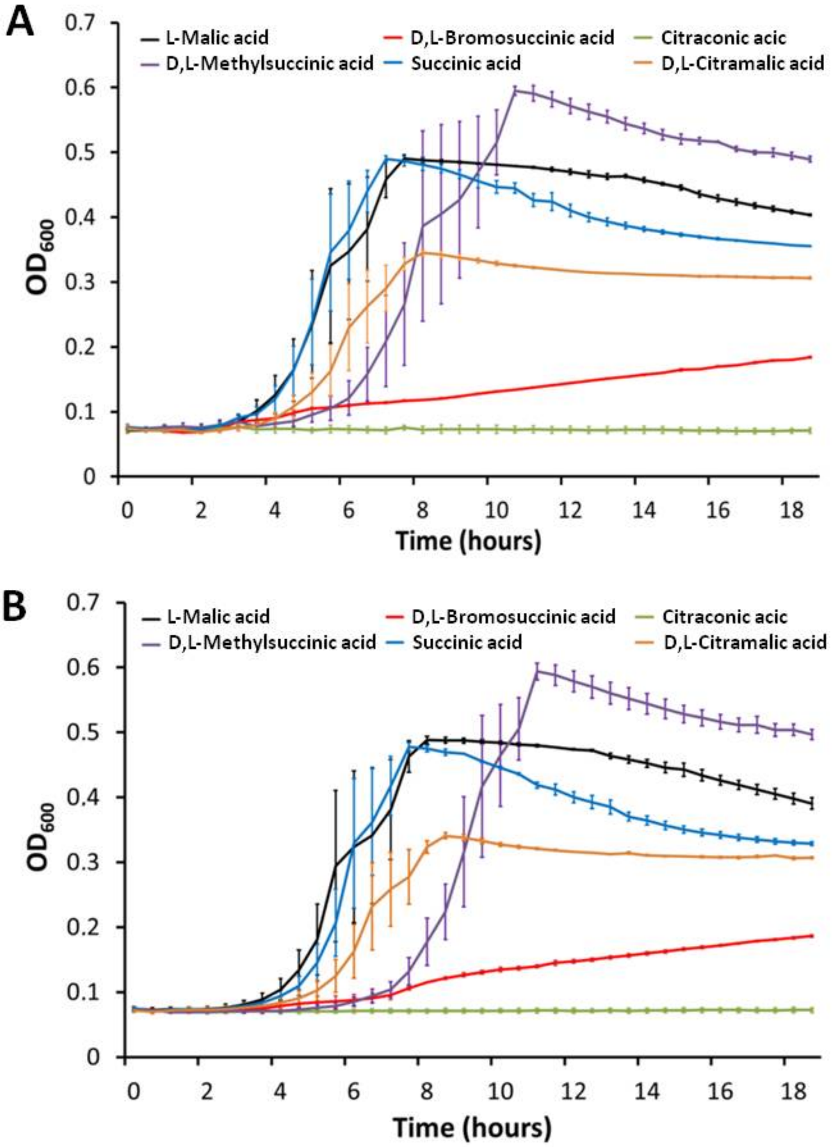

Supplementary Figure S5. Growth curves of *P. aeruginosa* PAO1 (A) and a mutant deficient in *PA2652* (B) in MS minimal medium supplemented with 5 mM of the different organic acids as sole carbon sources. Succinic acid was used an internal positive control. Data are means and standard deviations from three independent experiments.

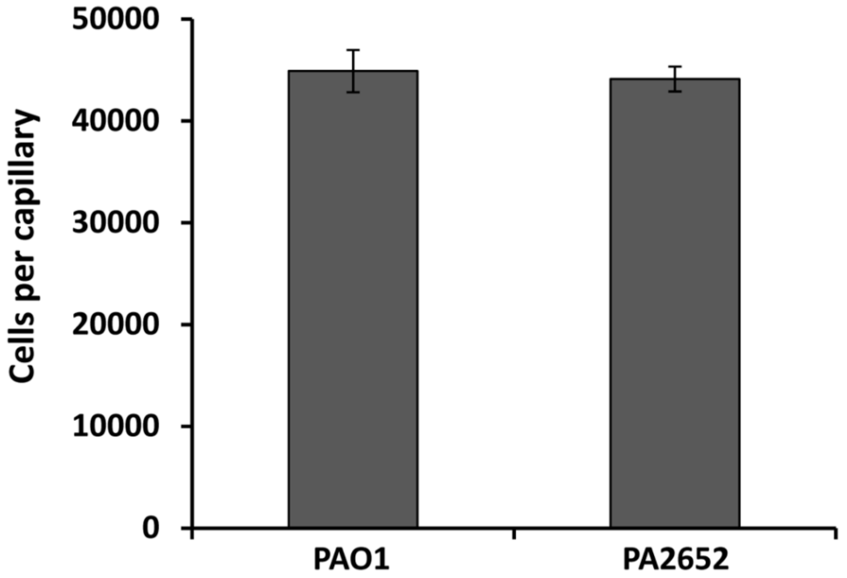

Supplementary Figure S6. Quantitative capillary chemotaxis assays of *Pseudomonas aeruginosa* PAO1 and its mutant in the *PA2652* gene towards 0.1 % (w/v) casamino acids. Data were corrected with the number of cells that swam into buffer containing capillaries (2376 ± 272). Data are the means and standard deviations from three biological replicates conducted in triplicate.

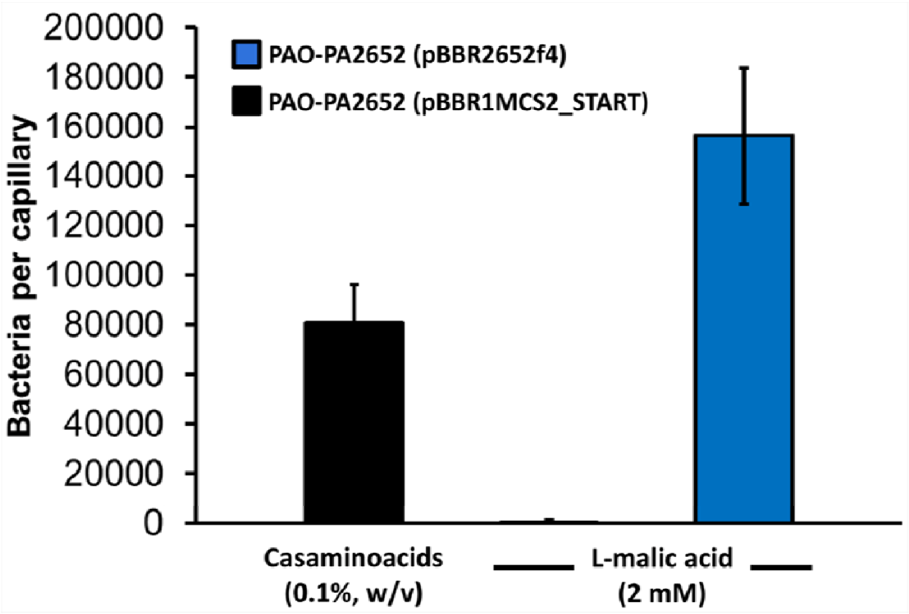

Supplementary Figure S7. Quantitative capillary chemotaxis assays showing the genetic complementation of a *Pseudomonas aeruginosa* PAO1 mutant strain defective in *PA2652*. Complementation assays of the *PA2652* mutant by the *in trans* expression of *PA2652* using the pBBRMCS2-based vector, pBBR2652f4. As positive control, chemotaxis toward casaminoacids of the mutant strain harboring the empty plasmid pBBRMCS2_START was analyzed. Data were corrected with the number of cells that swam into buffer containing capillaries (from left to right, 1333 ± 500, 3481 ± 755 and 4962 ± 1003). Data are the means and standard deviations from three biological replicates conducted in triplicate.

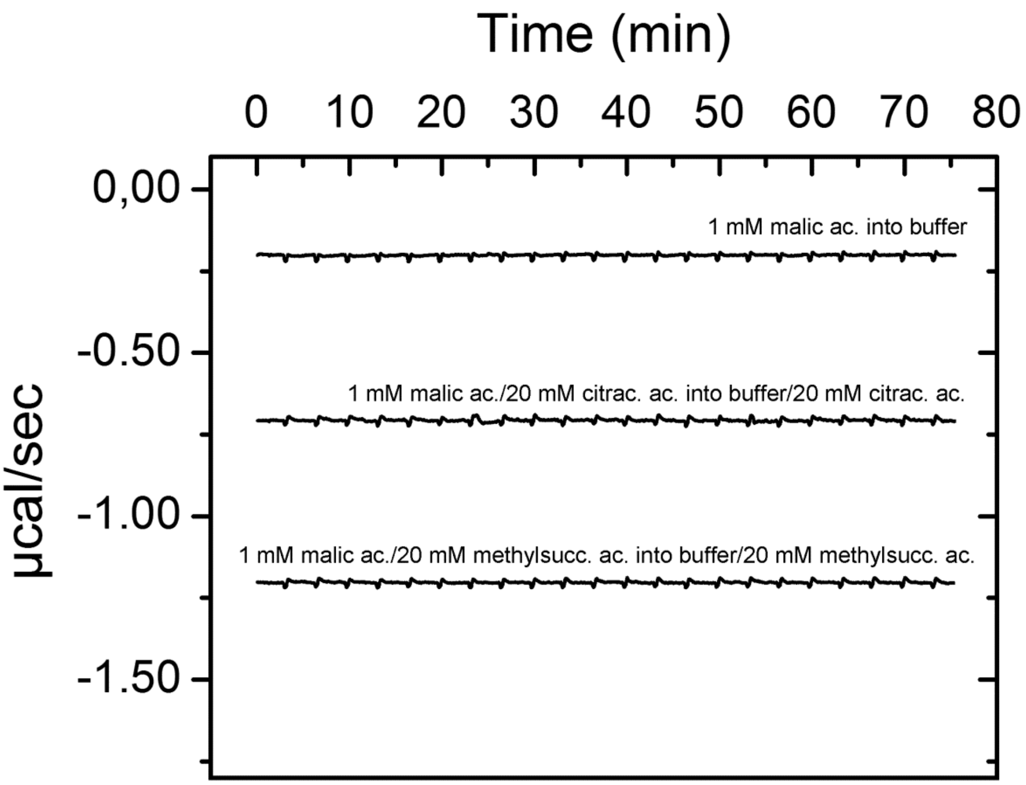

Supplementary Figure S8. Microcalorimetric titrations of buffer or buffer/antagonist mixtures with L-malic acid or L-malic acid/antagonist mixtures. In all cases the injection volume was of 9.6 µl. Data are the corresponding controls to experiments shown in Fig. 7.

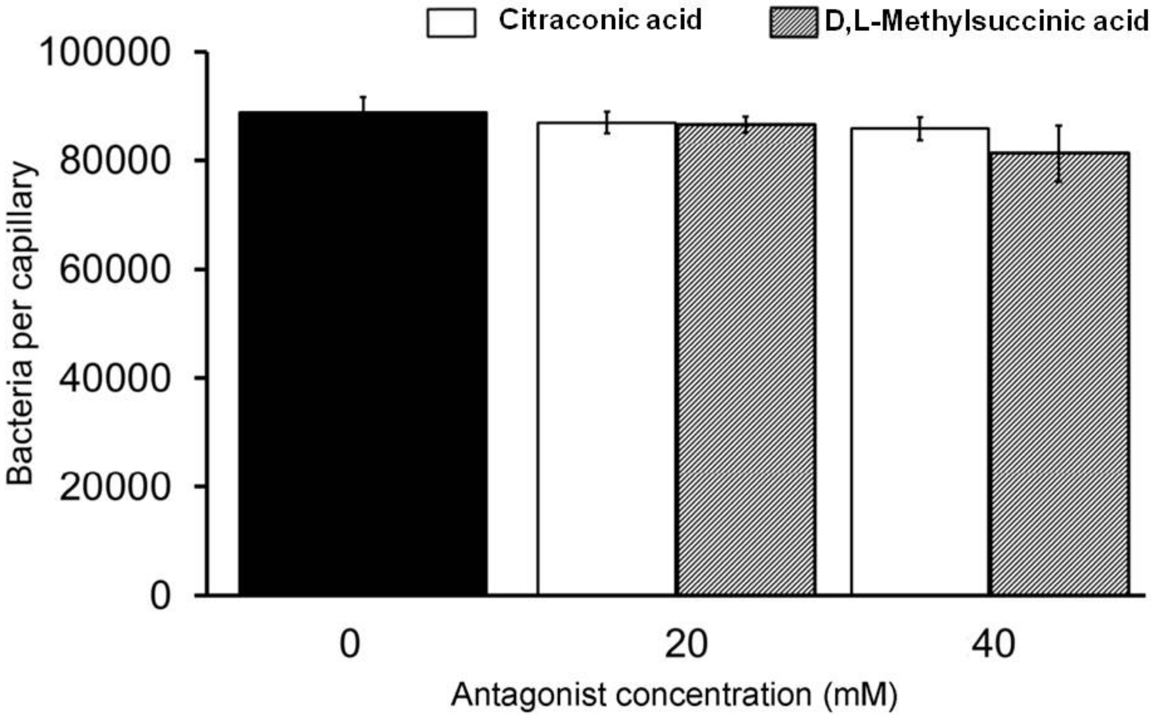

Supplementary Figure S9. Effect of citraconic and D,L-methylsuccinic acids in the chemotaxis properties of *Pseudomonas aeruginosa* toward L-alanine. Shown are number bacterial cells that migrate toward capillaries containing 1 mM of L-alanine (black bar) or 1 mM of L-alanine in the presence of different concentrations of citraconic and D,L-methylsuccinic acids. Data are means and standard deviations from three biological replicates conducted in triplicate. Data were corrected with the number of cells that swam into buffer containing capillaries (4400 ± 352).

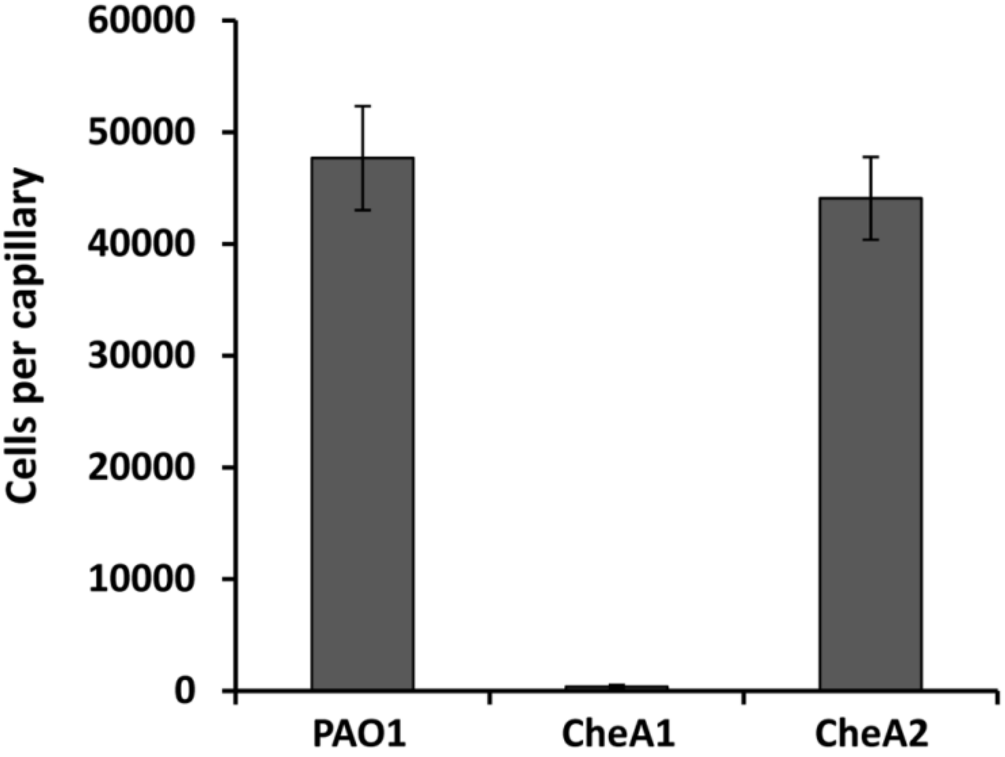

Supplementary Figure S10. Implication of *che* and *che2* chemosensory pathways in the chemotactic behavior of *Pseudomonas aeruginosa* PAO1 toward L-malic acid. Quantitative capillary chemotaxis assays were performed using L-malic acid at a final concentration of 10 mM. Data were corrected with the number of cells that swam into buffer containing capillaries (3115 ± 440). Data are the means and standard deviations from three biological replicates conducted in triplicate.

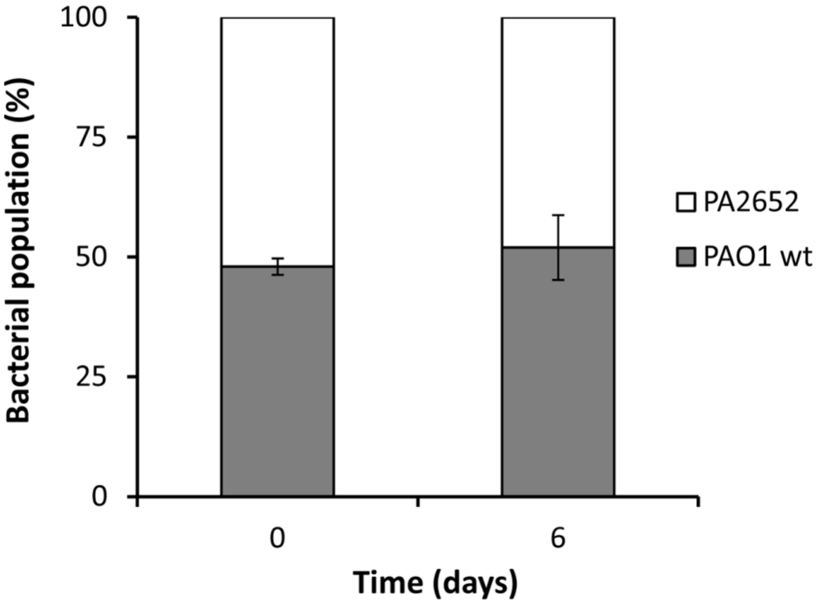

Supplementary Figure S11. Competitive root colonization of *Pseudomonas aeruginosa* PAO1-Km and a mutant defective in *PA2652*. The figure represents the percentage of bacteria recovered either from the rhizosphere or root tips of maize (*Zea mays*) plants. Data are the means and standard deviations of six plants.

Supplementary Table S1. Tm shifts of at least 2 ºC caused by the screening of compounds of Biolog arrays PM1, PM2A, PM3b, PM4a and PM5. The results of Isothermal Titration Calorimetry binding studies are also shown.

Compound	Tm Shift (ºC)	Binding in ITC
L-Malic acid	**+ 5.2**	**YES**
D,L-Bromosuccinic acid	**+ 3.6**	**YES**
Citraconic acid	**+ 2.5**	**YES**
D,L-Citramalic acid	**+ 2.1**	**YES**
D-Lactic acid methyl ester	**+ 2.1**	commercially not available
L-Pyroglutamic acid	**+ 2.0**	commercially not available
D,L-Methylsuccinic acid	Not in Biolog arrays	**YES**
D-Malic acid	No significant shift	NO
Succinic acid	No significant shift	NO
Fumaric acid	No significant shift	NO
Oxaloacetic acid	No significant shift	NO
L-Tartaric acid	No significant shift	NO
Glutaric acid	No significant shift	NO
L-Aspartic acid	No significant shift	NO
L-Threonine	No significant shift	NO

Supplementary Table S2. Apparent thermodynamic parameters derived from the microcalorimetric titrations of PA2652-LBD with L-malic acid in the absence and presence of the antagonists citraconic and methylsuccinic acids. Data were analysed using the "One binding site model" of the MicroCal version of ORIGIN. The corresponding data are shown in Fig. 7.

Antagonist	**n**	K_D (µM)	ΔH (kcal/mol)
none	1 ± 0.1	23 ± 1	-4.1 ± 0.6
2 mM citraconic acid	0.9 ± 0.2	53 ± 4	-5.1 ± 1
20 mM citraconic acid	1.1 ± 1	294 ± 58	-3.3 ± 5
2 mM D,L-methylsuccinic acid	0.6 ± 0.1	54 ± 5	-6.7 ± 1
20 mM D,L-methylsuccinic acid	1.1 ± 0.9	207 ± 31	-2.6 ± 2

Supplementary Table S3. Bacterial strains and plasmids used in this study.

Strain or plasmid	Relevant characteristics[a]	Reference or source
**Strains**		
Escherichia coli BL21 (DE3)	F^- *ompT gal dcm lon* $hsdS_B$ ($r_B^- m_B^-$) λ(DE3)	[4]
E. coli DH5α	*supE44 lacU169* (*Ø80lacZ*ΔM15) *hsdR17* ($r_k^- m_k^-$), *recA1 endA1 gyrA96 thi-1 relA1*	[5]
Pseudomonas aeruginosa PAO1	Wild type	[6]
PAO1-Km	wild type PAO1 with a Km cassette inserted in a neutral position downstream of *glmS*; Km^R	[7]
PAO-PA2652	*PA2652*::IS*phoA*/hah; Tc^R	[8,9]
PCheA1	PAO1 transposon mutant *PA1458*::IS*phoA*/hah; Tc^R	[8,9]
PCheA2	PAO1 transposon mutant *PA0178*::IS*lacZ*/hah; Tc^R	[8,9]
**Plasmids**		
pET28b(+)	Km^R; Protein expression plasmid	Novagen
pET28-PA2652-LBD	Km^R; pET28b(+) derivative containing DNA fragment encoding PA2652-LBD	This study
pBBR1MCS2_START	Km^R; *oriRK2 mobRK2*	[10]
pBBR2652f4	Km^R; *PA2652* gene was cloned into NdeI and BamHI sites of pBBR1MCS-2_START	This study

[a]The following abbreviations were used for antibiotics: kanamycin, km; tetracycline, Tc.

REFERENCES

1 Thompson, J. D., Higgins, D. G. & Gibson, T. J. CLUSTAL W: improving the sensitivity of progressive multiple sequence alignment through sequence weighting, position-specific gap penalties and weight matrix choice. *Nucleic Acids Res.* **22**, 4673-4680 (1994).

2 Combet, C., Blanchet, C., Geourjon, C. & Deleage, G. NPS@: network protein sequence analysis. *Trends Biochem. Sci.* **25**, 147-150 (2000).

3 Cserzo, M., Wallin, E., Simon, I., von Heijne, G. & Elofsson, A. Prediction of transmembrane alpha-helices in prokaryotic membrane proteins: the dense alignment surface method. *Protein Eng.* **10**, 673-676 (1997).

4 Jeong, H. *et al.* Genome sequences of *Escherichia coli* B strains REL606 and BL21(DE3). *J. Mol. Biol.* **394**, 644-652 (2009).

5 Woodcock, D. M. *et al.* Quantitative evaluation of *Escherichia coli* host strains for tolerance to cytosine methylation in plasmid and phage recombinants. *Nucleic Acids Res.* **17**, 3469-3478 (1989).

6 Stover, C. K. *et al.* Complete genome sequence of *Pseudomonas aeruginosa* PAO1, an opportunistic pathogen. *Nature* **406**, 959-964 (2000).

7 Martin-Mora, D. *et al.* Identification of a Chemoreceptor in *Pseudomonas aeruginosa* That Specifically Mediates Chemotaxis Toward alpha-Ketoglutarate. *Front. Microbiol.* **7**, 1937 (2016).

8 Jacobs, M. A. *et al.* Comprehensive transposon mutant library of *Pseudomonas aeruginosa*. *Proc. Natl. Acad. Sci. U.S.A.* **100**, 14339-14344 (2003).

9 Held, K., Ramage, E., Jacobs, M., Gallagher, L. & Manoil, C. Sequence-verified two-allele transposon mutant library for *Pseudomonas aeruginosa* PAO1. *J. Bacteriol.* **194**, 6387-6389 (2012).

10 Obranic, S., Babic, F. & Maravic-Vlahovicek, G. Improvement of pBBR1MCS plasmids, a very useful series of broad-host-range cloning vectors. *Plasmid* **70**, 263-267 (2013).
